# Identifying, Characterizing, and Engineering a Phenolic Acid-Responsive Transcriptional Factor from *Bacillus amyloliquefaciens*

**DOI:** 10.1021/acssynbio.3c00206

**Published:** 2023-07-27

**Authors:** Chenyi Li, Yuyang Zhou, Yusong Zou, Tian Jiang, Xinyu Gong, Yajun Yan

**Affiliations:** † School of Chemical, Materials and Biomedical Engineering, College of Engineering, The University of Georgia, Athens, Georgia 30602, United States

**Keywords:** PadR, transcriptional factors, biosensor engineering, *Bacillus amyloliquefaciens*

## Abstract

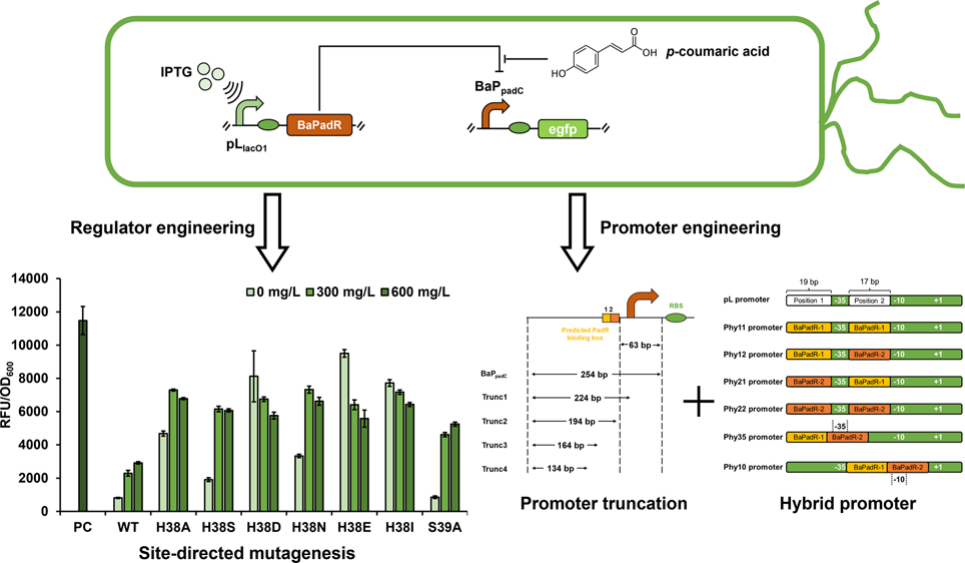

Transcriptional factors-based biosensors are commonly used in metabolic engineering for inducible control of gene expression and related applications such as high-throughput screening and dynamic pathway regulations. Mining for novel transcriptional factors is essential for expanding the usability of these toolsets. Here, we report the identification, characterization, and engineering of a phenolic acid responsive regulator PadR from *Bacillus amyloliquefaciens* (BaPadR). This BaPadR-based biosensor system showed a unique ligand preference and exhibited a high output strength comparable to that of commonly used inducible expression systems. Through engineering the DNA binding region of BaPadR, we further enhanced the dynamic range of the biosensor system. The DNA sequences that are responsible for BaPadR recognition were located by promoter truncation and hybrid promoter building. To further explore the tunability of the sensor system, base substitutions were performed on the BaPadR binding region of the phenolic acid decarboxylase promoter (P_padC_) and the hybrid promoter. This novel biosensor system can serve as a valuable tool in future synthetic biology applications.

## Introduction

Genetically encoded biosensors, such as allosteric transcriptional factors and riboswitches, are widely applied in metabolic engineering and synthetic biology.^1−3^ Among them, transcriptional factors-based biosensors are one of the most studied tools due to their great availability in nature and ease of manipulation and engineering.^4−6^

PadR is a phenolic acid responsive transcriptional repressor first discovered in *Bacillus subtilis.*(7−9) PadR can inhibit the promoter P_padC_ and repress the expression of the downstream *padC* gene, which encodes a phenolic acid decarboxylase. When the cells were exposed to an environment with an accumulation of phenolic acid, the phenolic acid would bind with the PadR and result in a conformational change of PadR, releasing the inhibition on *padC* transcription and activating the decarboxylation of phenolic acids.^7−9^ Due to the capability of sensing phenolic acids such as *p*-coumaric acid and ferulic acid, the important precursors for a series of valuable flavonoids and coumarins,^10−12^ this regulator was extensively studied and explored as a biosensor in metabolic engineering and synthetic biology.^9,13−15^ In 2017, the expression level of PadR was optimized in yeast through RBS engineering, and the engineered sensor system was used to screen high-producing strains of *p*-coumaric acid.^13^ In our previous studies, the PadR was further optimized with increased sensitivity, broader dynamic ranges, and expanded operational ranges.^9,15^ The engineered variants were then applied in establishing the dynamic pathway control to improve *p*-coumaric acid production^14^ and naringenin biosynthesis.^15^

The rapid development of advanced bioinformatic tools and fast-sequencing techniques has led to enormous amounts of genomic sequence data, which revealed a tremendous reservoir of putative transcriptional factors awaiting to be discovered.^16−18^ Here in this study, we identified a novel phenolic acid-responsive transcriptional regulator PadR from *Bacillus amyloliquefaciens* (BaPadR) through the protein sequence BLAST. Based on the knowledge of the previously characterized PadR from *Bacillus subtilis168* (BsPadR), we were able to locate the promoter BaP_padC_ that was controlled by the BaPadR and the DNA sequence in the promoter that was recognized by BaPadR. Further characterization of the BaPadR regulator revealed a unique ligand profile. To improve its usability as a biosensor system in metabolic engineering and synthetic biology, we expanded its dynamic ranges and increased its sensitivity through site-directed mutagenesis of the BaPadR regulator and base alteration on the BaPadR binding box. The BaPadR-based biosensor developed in this study expands the current repertoire of small-molecule-sensing transcriptional factors and can be a useful addition to the biosensor toolbox.

## Results

### Identifying a Potential Phenolic Acid-Responsive TF in *Bacillus amyloliquefaciens*

**Figure 1:**
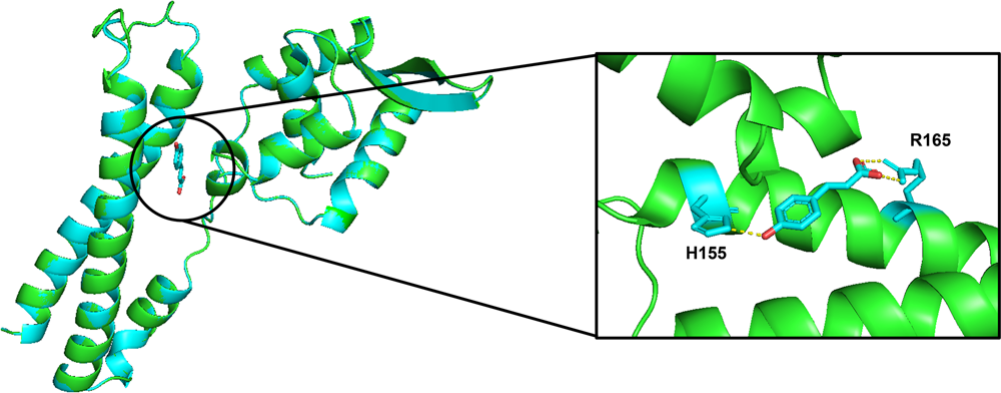
Simulation of BaPadR structure with *p*-coumaric acid binding based on the crystal protein structure of BssPadR (PDB: 5Y8T) using SWISS-Model. The green protein structure represents the BaPadR structure. The blue protein structure represents the BssPadR structure.

The protein sequence of the well-characterized PadR from *Bacillus subtilis168* (BsPadR) in our previous study^9^ was used as the template for BLAST (https://blast.ncbi.nlm.nih.gov/). A hit with 79.2% identity in the amino acid sequence was found (BAMF_RS24485, NCBI reference ID: WP_013351422.1) in *Bacillus amyloliquefaciens* ATCC 23350. Due to the high similarity in protein sequence, we believed this regulator possesses a matching function of BsPadR. While this protein (hereafter named with BaPadR) is annotated as a PadR family transcriptional regulator, its function has never been experimentally validated. A further pairwise sequence alignment revealed that most variations in BaPadR, compared to the BsPadR, are located in the regions of N75-D88 and A107-D145 (Figure S1a). As region A107-D145 overlaps the ligand binding pocket, we hypothesized that BaPadR might possess a different ligand spectrum or even a varied dynamic property. Since a nearly identical PadR from *Bacillus subtilissubsp. spizizenii str. W23* (BssPadR, with a sequence similarity of 89.6% compared to BaPadR) was previously crystallized and the structure information is available,^7^ the molecular model of BaPadR was built by employing Swiss-Model^19^ (https://swissmodel.expasy.org/) using the BssPadR (PDB ID: 5Y8T) as the template. The two proteins can be nearly perfectly aligned (Figure 1). The residues that interact with the native substrate (*p*-coumaric acid) were conserved and identified to be H155 and R165 in BaPadR (H154 and R164 for BsPadR and BssPadR) (Figure 1).

With the identification of BaPadR, we next sought to locate the promoter that is controlled by this regulator in *B. amyloliquefaciens*. As we know that the BsPadR controls the expression of the phenolic acid decarboxylase (PadC) in *B. subtilis*,^9^ we hypothesized that this was also the case in *B. amyloliquefaciens*. Thus, the protein sequence of PadC from *B. subtilis* (BsPadC) was used as the template for locating PadC in *B. amyloliquefaciens*. A hit (BAMF_RS37310, NCBI reference ID: WP_013353702.1) with 97.5% similarity was found and was believed to be the PadC of *B. amyloliquefaciens* (BaPadC) (Figure S1b). The upstream DNA sequence (700 bp) of the Ba*padC* gene was used to analyze the promoter location. Surprisingly, a short (429 bp) open reading frame (ORF) was discovered in this speculated promoter region (Figure S1c). As there were two short pseudogenes (*yveF* and *yveG*) in the composition of P_padC_ promoter in *B. subtilis* 168,^9^ we hypothesized this ORF encodes the homologous protein of YveG in *B. amyloliquefaciens*, based on the length of the ORF. A pairwise sequence alignment showed that there is a 55.6% similarity in protein sequence (Figure S1d), which was not significant enough to conclude that this was a YveG homologous protein. After a closer look of the sequence alignment results, we found that there was a 46-aa region at the N terminus of the BaYveG that has no match to the BsYveG (Figure S1d). Thus, we removed this sequence and realigned the protein sequence. The new sequence alignment revealed an 83.2% similarity in amino acid sequence (Figure S1e), which confirmed our hypothesis that this was likely the YveG homologous protein in *B. amyloliquefaciens*. Thus, the BaP_padC_ promoter was hypothesized to be the sequence before this *yveG* gene (Figure S1c).

### Reconstructing the BaPadR-BaP_padC_ Sensor System in *E. coli*

**Figure 2:**
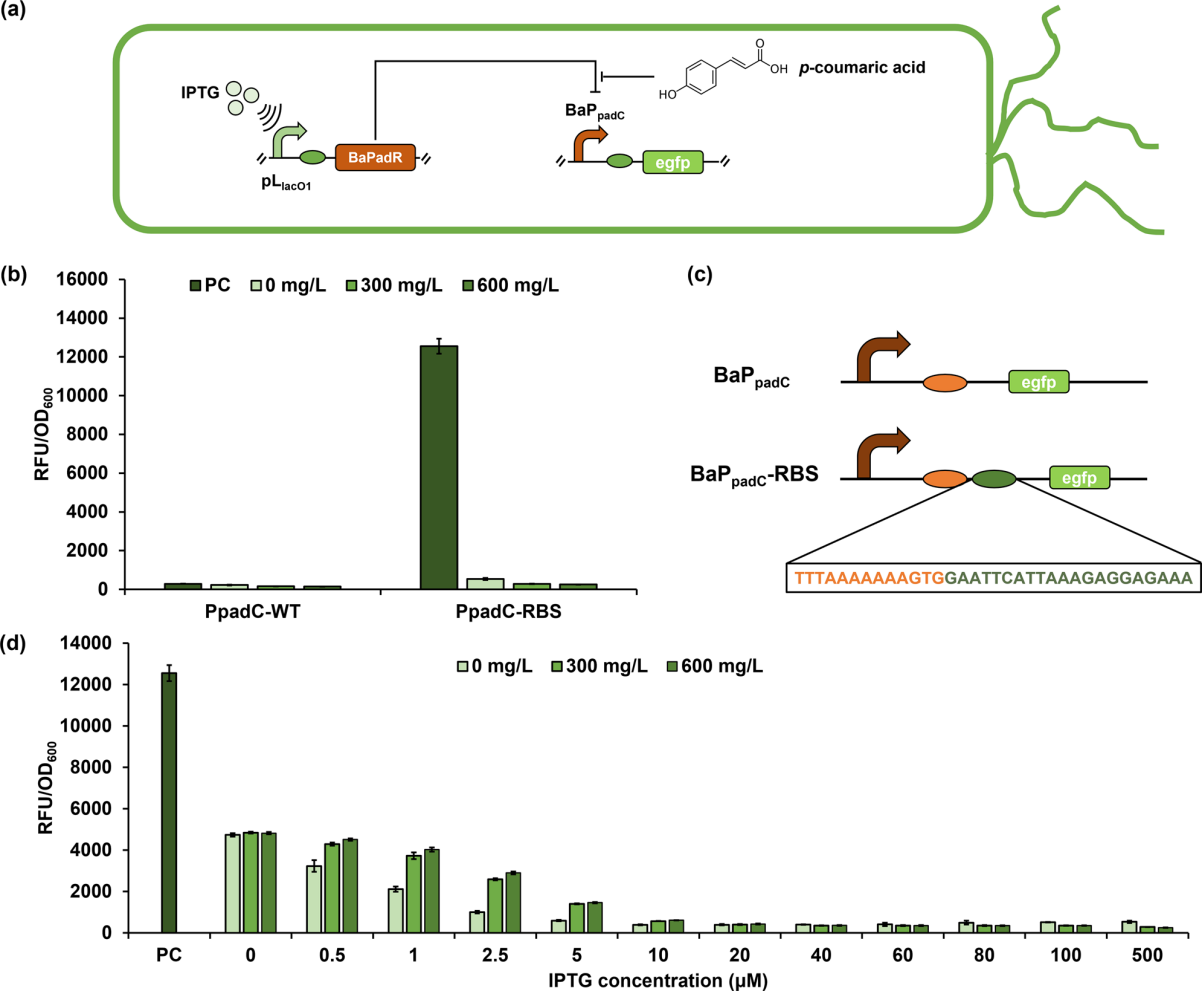
Reconstructing the BaPadR-BaP_padC_ sensor system in *E. coli*. (a) Schematic illustrations for how the PadR-PpadC from *Bacillus amyloliquefaciens* was reconstructed in *E. coli*. The BaPadR expression was controlled by IPTG-inducible promoter pL_lacO1_. The expression of BaPadR would inhibit the promoter activity of BaP_padC_ and thus repress the expression of egfp. The presence of *p*-coumaric acid would release such inhibition and recover the expression of egfp. (b) Dynamic performance of the WT BaP_padC_ promoter and the optimized BaP_padC_-RBS promoter. (c) Schematic illustration of how the engineered RBS was inserted. The brown sequence is the native promoter sequence. The orange sequence is the putative RBS sequence of the WT BaP_padC_ promoter. Between the native promoter sequence and the start codon of egfp, an additional RBS sequence (green) was inserted. (d) The dynamic performance of the reconstructed biosensor system in *E. coli*. BaP_padC_-WT represents the wild type BaP_padC_ promoter-controlled egfp expression cassette. BaP_padC_-RBS was the wild type BaP_padC_ promoter with the addition of an engineered RBS. PC represents the positive control without the BaPadR expression. All data represent the mean of 3 biologically independent samples, and error bars show standard deviation.

After the identification of BaPadR and the corresponding promoter BaP_padC_ that was controlled by this regulator, our next goal was to characterize the dynamic performance of this sensor system. Thus, we sought to re-establish this sensor system in the commonly used model chassis *Escherichia coli* (Figure 2a). To this end, the DNA sequence of BaPadR and the promoter BaP_padC_ was cloned from the genome of *B. amyloliquefaciens*. The BaPadR was placed under the control of the IPTG-inducible (IPTG was short for isopropyl β-d-1-thiogalactopyranoside) promoter pL_lacO1_ in the plasmid pCS27,^20^ and the BaP_padC_ promoter was used to control the expression of the reporter gene *egfp* in the plasmid pHA-egfp-MCS, resulting in pCS-BaPadR and pHA-BaP_padC_-WT-egfp. The plasmid pHA-BaPpadC-WT-egfp was cotransferred with pCS-BaPadR into *E. coli* BW25113 F′. An empty plasmid pCS27 without the expression of BaPadR was also cotransferred with the pHA-BaPpadC-WT-egfp to serve as the positive control (PC). To validate the function of this sensor system, we first chose the *p*-coumaric acid as the induction ligand because this compound was reported to be the effector for both BsPadR and BssPadR.^9^ Gradient concentrations of *p*-coumaric acid (0, 300, and 600 mg/L) were added into the cell culture to induce the sensor system, and 0.5 mM IPTG was added to induce the BaPadR expression. The green fluorescence intensity normalized, and cell density (OD_600_) was used to represent the promoter strength. Unexpectedly, the promoter exhibited very low activity (Figure 2b), and it did not show any responsiveness toward BaPadR or *p*-coumaric acid. We suspected that this was due to the low activity of the native RBS on the BaP_padC_ promoter. Thus, we added an additional strong RBS that was used in our previous studies^14^ between the BaP_padC_ promoter and the reporter gene *egfp* (Figure 2c). The new promoter configuration was constructed with the plasmid pHA-egfp-MCS, resulting in pHA-BaP_padC_-RBS-egfp, and the dynamic performance of the sensor system was tested. When no BaPadR was present (positive control), the promoter with the strong RBS (BaP_padC-RBS_) delivered a high output strength, with the normalized green fluorescence intensity reaching over 12,000 au. When the BaPadR expression was induced by 0.5 mM IPTG, the promoter activity drastically decreased by 95.7%, and only 539.16 au can be detected. However, further addition of *p*-coumaric acid did not relieve such inhibition. Instead, the promoter activity was further decreased (Figure 2b). We hypothesized this was likely due to the high expression level of the PadR, as this was also observed in our previous study in engineering the BsPadR.^9^ Thus, to reduce the BaPadR expression, we adjusted the activity of the pL_lacO1_ promoter by fine-tuning the inducing IPTG concentrations (Figure 2d). When the IPTG concentrations were decreased to 10 μM, the biosensor system showed a response toward *p*-coumaric acid. Further decrease of the IPTG concentration to 0.5 μM enhanced this responsiveness, but the leaky activity of the promoter (when no *p*-coumaric acid was added) also increased (Figure 2d). These results demonstrated the successful reconstruction of this sensor system and validated the function of the BaPadR. One notable feature of the BaPadR-based biosensor system is its high inhibition efficiency. For example, when the IPTG concentration was set to 2.5 μM (which is 200-fold lower than the normal induction concentration of 0.5 mM), the BaPadR still showed a high inhibition efficiency toward the BaP_padC_ promoter even though it was expressed from a medium-copy plasmid (while the BaP_padC_ promoter was placed on the high-copy plasmid). We also noticed that there was a decrease in *egfp* expression level when the pCS-BaPadR plasmid was further introduced to the PC (group IPTG = 0 in Figure 2d). This was likely due to the burden of the two-plasmid system, which could reduce the cell performance and decrease the protein expression. Compared to the control without any induction of BaPadR expression (IPTG = 0), 600 mg/L *p*-coumaric acid can release nearly 60% inhibition while still maintaining a low leaky activity when the IPTG concentration was set to 2.5 μM. Thus, this IPTG concentration was selected to induce the BaPadR expression in our following experiments.

### Substrate Scope of the Reconstructed BaPadR-BaP_padC_ Sensor System

After the successful establishment of the BaPadR-BaP_padC_ sensor system with a usable dynamic performance in *E. coli*, our next goal was to profile the ligand scope of this sensor system. Since we observed a varied amino sequence in the substrate binding region (from A107-D145) of BaPadR compared to BsPadR (Figure S1a), we anticipated that BaPadR would possess a different ligand spectrum.

**Figure 3:**
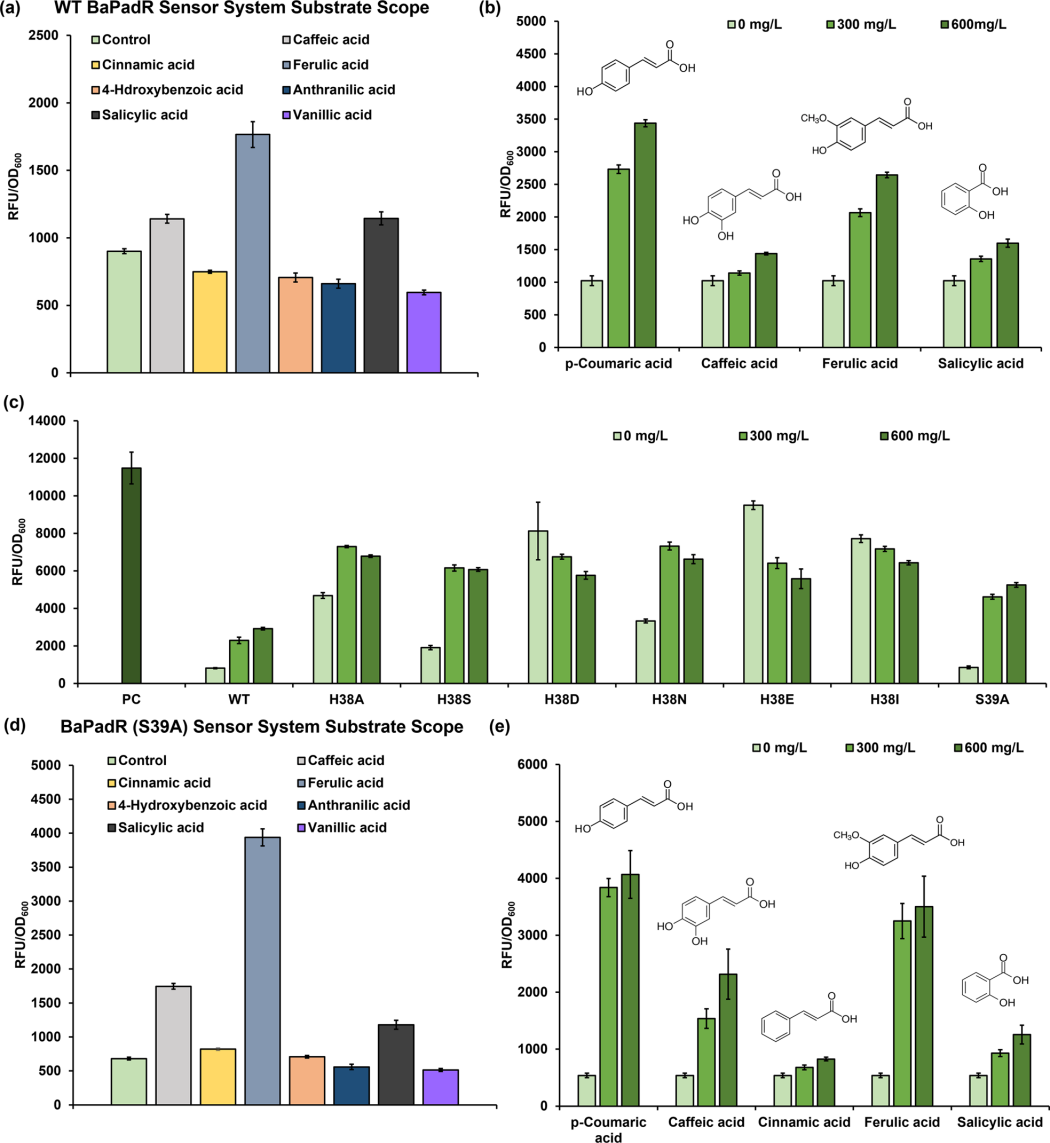
Profiling the ligand scope of the reconstructed BaPadR-BaP_padC_ sensor system, and site-directed mutagenesis to weaken the BaPadR binding affinity. (a) Screening the potential substrates of BaPadR. The concentration of the effectors was 300 mg/L in this experiment. (b) Dynamic performance of the BaPadR toward *p*-coumaric acid, caffeic acid, ferulic acid, and salicylic acid. (c) The dynamic performance of the BaPadR variants under the induction of 2.5 μM IPTG. (d) Screening of the potential substrates of the BaPadR (S39A) variant. The concentration of the effectors was 300 mg/L in this experiment. (e) Dynamic performance of the BaPadR (S39A) variant toward *p*-coumaric acid, caffeic acid, cinnamic acid, ferulic acid, and salicylic acid. All data represent the mean of 3 biologically independent samples and error bars show standard deviation.

As PadR was normally considered as the phenolic acid responsive regulator, we tested its responsiveness against three phenolic acids and four smaller benzoic acid derivatives (Figure 3a). These aromatic compounds are important precursors for value-added flavonoids,^21^ alkaloids,^22^ and coumarins.^23^ Compared with the uninduced control, the caffeic acid, ferulic acid, and salicylic acid (with a concentration of 300 mg/L for each compound) enabled an observable increase of egfp expression level upon the induction, which indicated potential responsiveness of BaPadR toward these chemicals (Figure 3a). Thus, we further tested the dynamic ranges of the three additional substrates in addition to the *p*-coumaric acid (Figure 3b). The best effector was determined to be *p*-coumaric acid, which resulted in a 3.36-fold increase of the *egfp* expression level upon induction by a concentration of 600 mg/L, followed by ferulic acid (2.58-fold), salicylic acid (1.56-fold), and caffeic acid (1.40-fold), respectively. The induction by salicylic acid was unexpected as it is much smaller than the other responsive phenolic acid and lacks the hydroxy group at the *para* position, which we thought is critical for substrate recognition of the PadR regulator.^15^

### Site-Directed Mutagenesis to Weaken the BaPadR Binding Affinity

While the reconstructed BaPadR-BaP_padC_ biosensor system shows a usable dynamic performance, inhibition of the BaPadR on the BaP_padC_ promoter cannot be fully released. Although through weakening the BaPadR expression can increase the output activity of the sensor system, it will also result in high leaky activity which would reduce the induction fold. We suspected that the high inhibition efficiency of BaPadR was likely due to the high binding affinity between BaPadR and the promoter BaP_padC_. While this would benefit the cell with less metabolic burdens (other sensor systems often require higher expression levels of regulator to achieve the regulation), the dynamic range of this biosensor system was restricted by such high affinity. Thus, we sought to investigate the DNA binding region of BaPadR, aiming to weaken the binding affinity between BaPadR and the promoter BaP_padC_, which, we believe, can further increase the dynamic range and result in more variants of the sensor system.

In our previous study,^9^ engineering the DNA binding region of BsPadR enabled a better dynamic performance of the BsPadR-based biosensor system. The two highest increases in output strength resulted from mutating the H38 (over 3-fold) or S39 (∼1.6-fold) to alanine in BsPadR, respectively, which reduced the binding affinity between BsPadR and its corresponding promoter P_padC._^9^ As the BaPadR possesses a nearly identical amino acid sequence in the DNA binding region compared to BsPadR (Figure S1a), we hypothesized that this would be the same case for engineering BaPadR. Thus, two target critical amino acids were selected to be H38 and S39 in BaPadR. Since the most prominent increase in the dynamic response was from mutating the H38 in BsPadR, we believe this amino acid would also be the dominant one in mediating the binding between BaPadR and BaP_padC_. Rather than directly mutating the big histidine residue to the small alanine, we wanted to investigate how the change of the amino acid residue size can affect the DNA binding of BaPadR. To this end, we designed and constructed six BaPadR mutants (H38I, H38E, H38N, H38D, H38S, and H38A). Along with these six variants, the BaPadR (S39A) was also included in the dynamic performance test as it was also demonstrated to be beneficial for weakening the DNA binding in BsPadR.^9^ As we expected, mutating H38 to smaller amino acids, such as H38A, H38S, and H38N, can enhance the dynamic response of the BaPadR-based biosensor system, but they also enabled increased leaky activities (Figure 3c). Surprisingly, variants H38D, H38E, and H38I abolished the BaPadR-based regulation, resulting in no response to increased *p*-coumaric acid concentrations (Figure 3c). We suspected this was likely because the change of amino acid altered the structure of the DNA binding region, which disabled the binding of BaPadR toward the promoter. The best performer was the S39A variant, which led to a 1.80-fold increase in the dynamic response but did not increase the leaky expression of the biosensor system. With the S39A variant, the BaPadR-BaP_padC_ biosensor system enabled a 1.71-fold increase in the dynamic range. The enhanced dynamic range of the biosensor would be more practical for metabolic engineering applications, such as dynamic pathway regulation and high-throughput screening.

Since the modification of the amino acid may alter the regulator protein structure, it is likely that the inducer scope of the BaPadR variant would also be changed. Thus, we tested the substrate scope of the best-performing BaPadR (S39A) variant. By following the previous procedure for testing the inducer scope of the wild-type BaPadR, 300 mg/L of different ligands was added to test the initial responsiveness. Compared with the control group, caffeic acid, ferulic acid, and salicylic acid induced a visible increase in *egfp* expression as well (Figure 3d). Surprisingly, cinnamic acid, which also lacks the hydroxy group at the para position, could slightly release the inhibition from the BaPadR (S39A) variant (Figure 3d). We further applied a gradient concentration of those chemicals plus *p*-coumaric acid to induce the BaPadR (S39A)-BaP_padC_ sensor system (Figure 3e). *p*-Coumaric acid still performed best to activate the *egfp* expression, which resulted in a 7.55-fold increase of the *egfp* expression level under 600 mg/L inducer concentration, followed by ferulic acid (6.50-fold), caffeic acid (4.30-fold), salicylic acid (2.33-fold), and cinnamic acid (1.53-fold), respectively. Notably, compared to WT BaPadR, all responsive substrates showed increased dynamic ranges when inducing the new variant BaPadR (S39A).

### Identifying a Potential PadR Binding Box via Promoter Truncation and Hybrid Promoter Construction

**Figure 4:**
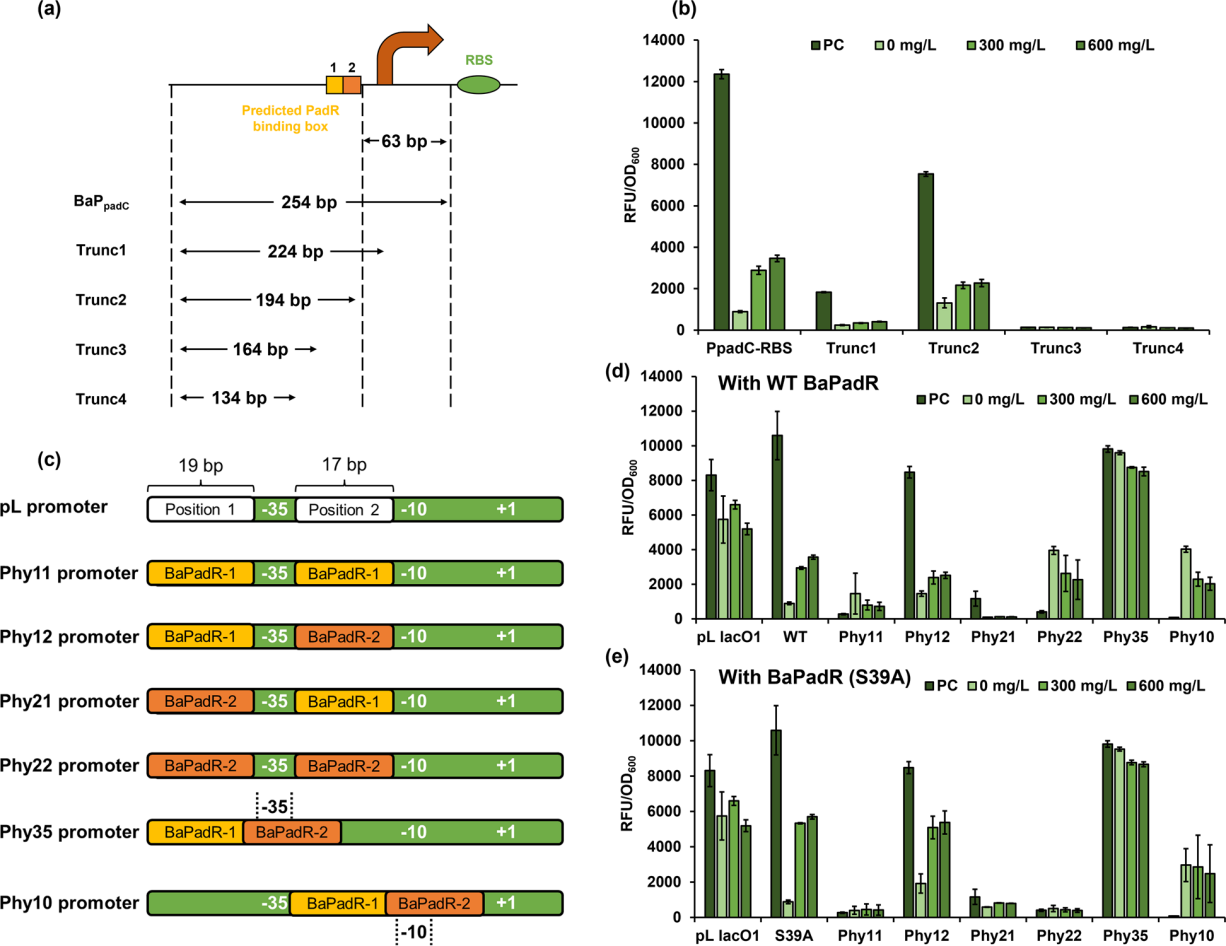
Identification of potential PadR binding box in the BaPpadC promoter and hybrid promoter construction. (a) Schematic illustrations for the truncation of BaP_padC_ promoter. (b) Dynamic performance of the truncated promoters. (c) Schematic illustration of the hybrid promoter design strategy. (d) Dynamic performance of the hybrid promoters with WT BaPadR. (e) Dynamic performance of the hybrid promoters with BaPadR (S39A). All data represent the mean of 3 biologically independent samples and error bars show standard deviation.

With the optimized performance of the BaPadR-BaP_padC_ sensor system, our next goal is to identify the DNA sequence that is responsible for BaPadR binding in the BaP_padC_ promoter. After a close investigation of the BaP_padC_ promoter sequence, two 18-bp sequences in the promoter region were identified (Figure S1c): one is “-CATGTAAATAGTTACATG-” that was identical with the BsPadR binding sequence,^9^ and the other one is “-CATGTATATATAAACATA-” which shows 5 mismatches but is still highly similar to the BsPadR binding sequence.^7,9^ These two units were also overlapped in the BaP_padC_ promoter region (Figure S1c), in which a similar distribution of binding box was observed in the BsP_padC_ promoter. Thus, we hypothesized that these two sequences are the target recognition sites for BaPadR binding. To test this hypothesis, we first designed a promoter truncation experiment. By truncating the promoter and eliminating the potential binding sequence, we want to see whether the promoter can still respond to BaPadR and *p*-coumaric acid. Thus, four truncated promoters (Trunc1–4) were designed (Figure 4a). Every truncation, from Trunc1 to Trunc4, would shorten the promoter by 30 bp from 3′ end (Figure 4a). Notably, the Trunc2 promoter would eliminate the second BaPadR binding box (BaPadR-2, with the sequence “-CATGTATATATAAACATA-”) but keep the first BaPadR binding box (BaPadR-1, with the sequence “-CATGTAAATAGTTACATG-”). The promoters Trunc3 and Trunc4 no longer possess the suspected BaPadR binding boxes. After promoter truncation, we tested the dynamic performance of the biosensor system with the four truncated promoters. As expected, the promoters Trunc3 and Trun4 lost most of the promoter activities and cannot be repressed by BaPadR (Figure 4b). On the contrary, the promoter Trunc1 can still be repressed by BaPadR and activated by p-coumaric acid, albeit with a low promoter activity (Figure 4b). Surprisingly, the promoter Trunc2, with only BaPadR-1, can still be inhibited by BaPadR and then be activated by adding *p*-coumaric acid to the media (Figure 4b). This might indicate that even with only the BaPadR-1 binding box present, the BaPadR can still recognize the sequence and bind to the promoter. The observation that Trunc2 exhibits overall higher activity than Trunc1 (Figure 4b) may stem from intrinsic differences in the promoter architecture, such as the relative arrangement and spacing of functional motifs within the promoter region. It is also likely that the absence of box 2 in the Trunc2 promoter contributes to the higher activities. In summary, the promoter truncation experiment strongly suggested that these two DNA sequences are responsible for BaPadR recognition.

To further validate the function of the BaPadR binding boxes and to investigate whether these two boxes can be used in a “plug-and-play” manner, we sought to design hybrid promoters by inserting the binding boxes into a promoter that cannot be regulated by BaPadR or *p*-coumaric acid. The commonly used pL_lacO1_ promoter was selected in our study. Previously, Lutz and colleagues inserted two LacO binding boxes into the position 1 and position 2 (Figure 4c) and converted the constitutive promoter pL to the commonly used inducible promoters pL_lacO1._^24^ We aimed to follow this strategy by placing the BaPadR binding boxes into these two positions. We hypothesized that substituting the LacO binding boxes in positions 1 and 2 with the BaPadR binding boxes would allow the BaPadR to recognize the hybrid promoters and inhibit the transcription of downstream reporter gene. Thus, a total of 4 hybrid promoters (Phy11, Phy12, Phy21, and Phy22) were designed by substituting the two binding boxes at different positions (Figure 4c). Because there was only 17 bp between the −35 and −10 regions of the pL promoter, when replacing the promoter sequence in this region, the last base pair of the BaPadR binding box was deleted to keep an intact −10 region. Considering that the two BaPadR binding boxes were overlapped in the original promoter, we also wanted to explore how the hybrid promoters would function if the BaPadR binding boxes were kept at the original form. Thus, we placed the original sequence of the BaPadR binding box (a 32-bp-long sequence, Figure S1c) to overlap with either the −35 region or −10 region of the promoter, aiming of guiding the BaPadR to bind with these two regions and interfere the RNA polymerase binding. As a result, two additional hybrid promoters (Phy35 and Phy10) were designed (Figure 4c). When overlapping with the −35 or −10 region, the corresponding base pairs in the BaPadR binding boxes were deleted to keep the intact −35 or −10 region. These six hybrid promoters were constructed to the pHA-egfp-MCS plasmid to control the expression of the reporter gene *egfp*. The dynamic performance of the biosensor system harboring these six hybrid promoters was evaluated with the WT PadR (Figure 4d). The original pL_lacO1_ promoter was also included as the control. The dynamic performance test validated that the original pL_lacO1_ promoter cannot be regulated by BaPadR or *p*-coumaric acid (Figure 4d). Notably, the BaP_padC_ promoter showed higher activity than the pL_lacO1_ promoter, indicating its great potential in applications in synthetic biology and metabolic engineering (Figure 4d). Among the six hybrid promoters, Phy12 maintained a promoter activity comparable to that of pL_lacO1_, and about 82.8% promoter activity of Phy12 can be inhibited by BaPadR. After addition of 600 mg/L *p*-coumaric acid, around 29.7% promoter activity can be recovered (Figure 4d). The Phy21 promoter, while only exhibiting a low promoter activity, can also be inhibited by the BaPadR (with an inhibition efficiency of 91.7%), but only 10.2% promoter activity can be recovered by 600 mg/L *p*-coumaric acid. Except Phy12 and Phy21, the remaining four hybrid promoters did not show any inhibition when BaPadR was present. As the BaPadR (S39A) variant showed a better dynamic performance compared to that of WT BaPadR, we also tested the dynamic performance of the six hybrid promoters against the S39A variant (Figure 4e). The inhibition efficiencies of BaPadR on the Phy12 and Phy21 promoter were changed to 77.4% and 49.2%, respectively, but the response of these two promoters toward *p*-coumaric acid was improved. Around 63.4% and 68.5% of promoter activities can be recovered by inducing 600 mg/L *p*-coumaric acid for Phy12 and Phy21, respectively. The dynamic performance results of Phy12 and Phy21 confirmed that the suspected binding boxes are critical for BaPadR recognition and inhibition and that the two binding boxes can be used for constructing hybrid promoters that can be regulated by BaPadR.

### Single Base Replacement on the Promoter to Further Weaken the BaPadR Binding Affinity

As the BaPadR binding boxes have been characterized, we aimed to further reduce the binding affinity between the regulator and the promoter and to investigate the impact of the nucleotide alteration in the binding box on the output of the entire sensor system.

Upon further investigation of the promoter truncation results, we assumed that the BaPadR-1 sequence (“-CATGTAAATAGTTACATG-”) was the primary sequence recognized and bound by BaPadR, because the Trunc2 promoter, which only possessed the BaPadR-1 sequence, can still be controlled by BaPadR and *p*-coumaric acid (Figure 4b). Thereby, we decided to replace the key bases in the BaPadR-1 binding box and to investigate the impacts on dynamic performance of the sensor system. Additionally, as revealed in a previous study,^7^ in the BsPadR-BsP_padC_ binding structure, the BsPadR makes direct contact with the bases on C6, T8, G9, T10, T17′, and T18′ sites (primes (′) indicate the bases are on the complementary strand). Based on the previous research,^7^ the substitutions at positions C6, T8, and T18′ led to obvious reduction in the binding affinity of BsPadR-BsP_padC_, with the degree of reduction ranging from 1-fold to nearly 40-fold.^7^ Since the BaPadR-1 sequence is completely identical with the BsPadR binding sequence, we hypothesized that these three positions (C6, T8, and T18′) also played a crucial role in the binding of BaPadR and the promoter. Therefore, we introduced base substitution in the BaPadR-1 binding box at the C6, T8, and T18′. Based on the BaP_padC_-RBS promoter, several base-replaced promoters were obtained, including promoter P_padC_-C6A-RBS, P_padC_-C6T-RBS, P_padC_-C6G-RBS, P_padC_-T8A-RBS, P_padC_-T8C-RBS, P_padC_-T8G-RBS, P_padC_-T18′A-RBS, P_padC_-T18′C-RBS, and P_padC_-T18′G-RBS. We also performed base alteration on the well-performed hybrid promoter Phy12, resulting in promoters Phy12-C6A, Phy12-C6T, Phy12-C6G, Phy12-T8A, Phy12-T8C, Phy12-T8G, Phy12-T18′A, Phy12-T18′C, and Phy12-T18′G. All those promoter variants were constructed on plasmid pHA-egfp-MCS and carried the expression of the reporter gene *egfp*.

**Figure 5:**
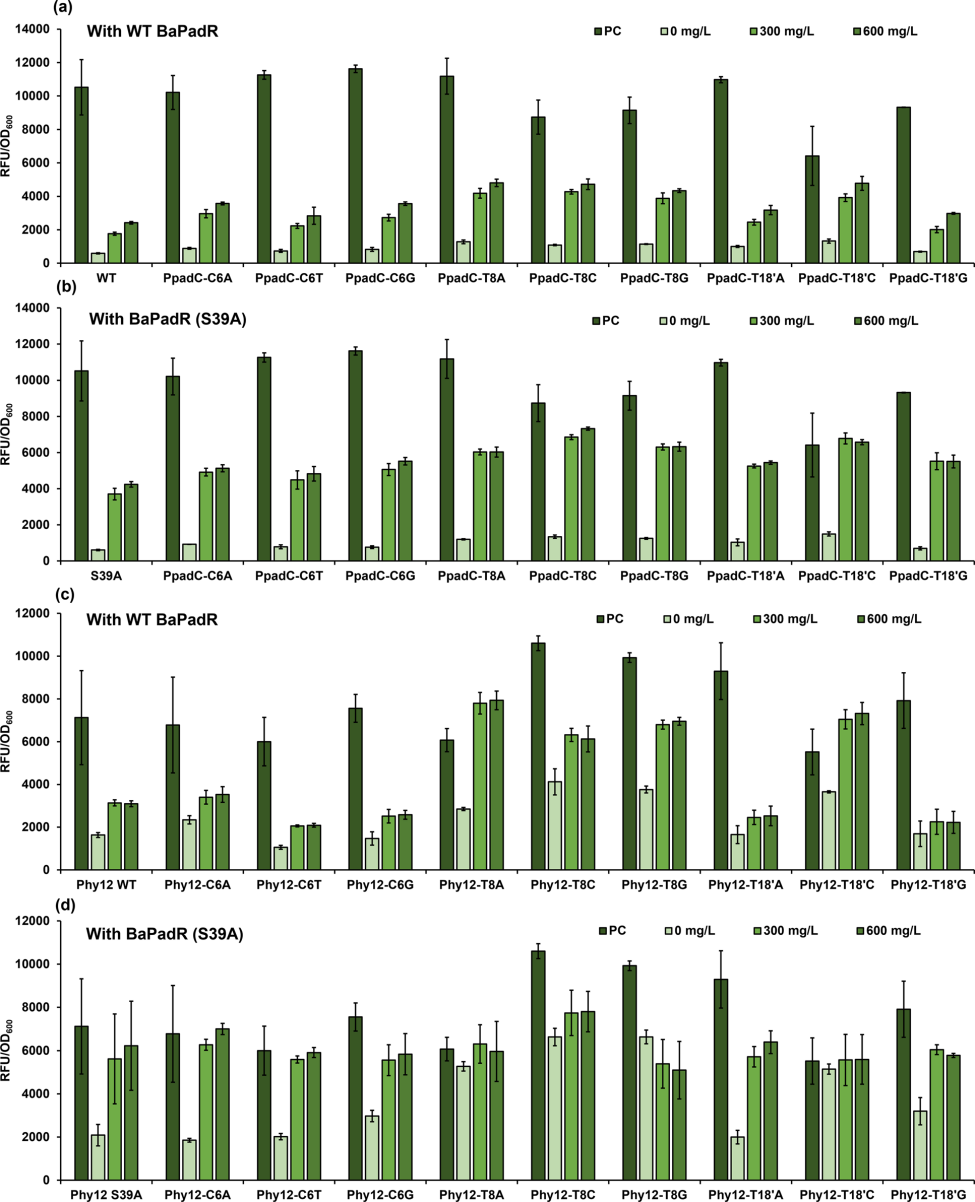
Exploring the influence of base substitution on the dynamic performance. (a) Dynamic performance of the PpadC promoter and its variants controlled with WT BaPadR. (b) Dynamic performance of the PpadC promoter and its variants controlled with the BaPadR-S39A variant. (c) Dynamic performance of the hybrid promoter Phy12 and its variants was controlled with WT BaPadR. (d) Dynamic performance of the hybrid promoter Phy12 and its variants controlled with the BaPadR-S39A variant. All data represent the mean of 3 biologically independent samples and error bars show standard deviation.

The dynamic performance of the base-substituted P_padC_ promoter variants was tested. The original P_padC_ promoter was included as a control. With WT BaPadR, the change of C6 nucleotide did not significantly reduce the binding affinity compared to the original P_padC_ promoter (Figure 5a). The inhibition efficiency was further decreased when the base substitution happened on the T8 site (Figure 5a). The promoter P_padC_-T8A exhibited the highest output strength under 600 mg/L *p*-coumaric acid, which was 4,806.20 au. Mutations at the T18′ position resulted in a more complex dynamic range change scenario. The P_padC_-T18′A exhibited a similar dynamic performance compared to the original P_padC_ promoter (Figure 5a). We noticed that the alteration from thymine (T) to cytosine (C) on the T18′ site severely impacted the promoter activity, which resulted in a 39.0% promoter activity reduction (Figure 5a). However, 61.0% promoter activity of P_padC_-T18′C could be released with 600 mg/L *p*-coumaric acid (Figure 5a). A similar trend by base alteration can be observed when the sensor systems were controlled by BaPadR (S39A) compared with that by WT BaPadR, but the dynamic performance of each promoter was enhanced when the S39A variant was employed (Figure 5b). Although the inhibition efficiency by the S39A variant toward the P_padC_-C6A, P_padC_-C6T, and P_padC_-C6G slightly reduced, the activation strength by 600 mg/L *p*-coumaric acid increased 43.5%, 70.0%, and 55.0%, respectively (Figure 5b). Notably, the inhibition from the S39A variant toward the P_padC_-T18′C promoter could be fully released by only 300 mg/L *p*-coumaric acid (Figure 5b).

The dynamic performance of all of the Phy12 promoter variants was also tested. Similar to the situation in the P_padC_ promoter and its variants, the mutations in the C6 position did not significantly influence the dynamic performance of the WT BaPadR-Pp_adC_ sensor system (Figure 5c). However, when the S39A variant was performed, the promoter activity of Phy12-C6A and Phy12-C6G could be fully recovered with 600 mg/L *p*-coumaric acid (Figure 5d). The mutation on T8 site significantly weakened the inhibition ability of the BaPadR (Figure 5c and 5d). Due to the extremely low inhibition efficiency, all the Phy12-T8 promoter mutants almost lost their regulatory ability by BaPadR (S39A) and *p*-coumaric acid (Figure 5d). When the base substitution occurred on the T18′ site, the Phy12-T18′A and Phy12-T18′C variant showed a similar dynamic range compared with the original Phy12 promoter (Figure 5c and 5d). Similar to what we observed in the P_padC_ promoter, the Phy12-T18′C activity was negatively affected (Figure 5c), along with a narrowed dynamic range. Combining with the results we observed in the BaP_padC_ promoter, this may indicate that the substitution from T to C in the T18′ position not only affects the binding affinity of BaPadR to the promoter but also directly caused a negative impact on the intrinsic activity of the promoter.

In summary, through nucleotide substitutions, we obtained several engineered promoters that showed enhanced responsiveness while still maintaining relatively strict efficiencies, such as P_padC_-C6A, P_padC_-T8A, and P_padC_-T8G. These base-replacement promoters greatly expanded the reservoir of the phenolic acid-responsive biosensor system, and they can be readily used in biosensor-enabled metabolic engineering applications.

## Discussion

Transcriptional factors-based biosensors have become increasingly important in establishing efficient microbial cell factories for biosynthesis of valuable compounds. Due to their ability to sense small molecules or environmental signals in a real-time manner, a series of biosensor-enabled strategies have been developed, such as dynamic pathway regulations and high-throughput screenings. To expand the applicability of these strategies, the current repertoire of transcription-factor-based biosensors needs to be widened. Here, we identified, characterized, and engineered a novel phenolic acid responsive regulator from *Bacillus amyloliquefaciens* (BaPadR). Through sequence alignment, we located the promoter regulated by the BaPadR which controls the expression of a phenolic acid decarboxylase (the promoter was then named as BaP_padC_). The protein sequence variations in the ligand binding domain inspired us to further characterize this sensor system. Re-establishing the biosensor system in *E. coli* confirmed the function of the BaPadR and corresponding promoter BaP_padC_. Notably, the output strength of this reconstructed biosensor system is comparable to a commonly used inducible system and the BaPadR exhibited a unique activity to cinnamic acid and salicylic acid, which was not observed in previously reported PadR regulators.^9^ To further enhance the dynamic response of the biosensor system, we investigated the DNA binding of BaPadR and performed site-directed mutagenesis to diminish the binding affinity between BaPadR and BaP_padC_. Through sequence alignment and promoter truncation, we located the binding boxes in the promoter that are responsible for BaPadR recognition. Further construction of hybrid promoters using the binding boxes confirmed its function and demonstrated a “plug-and-play” feature for these DNA sequences. The base substitution on the binding box of the promoter increased the dynamic performance diversity of the BaPadR-based sensor system. Compared to previously identified PadR regulators, BaPadR demonstrated a distinctive response to the smaller salicylic acid, while retaining its ability to bind with phenolic acids. Through the engineering efforts in this study, we successfully obtained several promoters (e.g., PpadC-T18′C, Phy12-C6A, and Phy12-C6T) that exhibited full induction by 600 mg/L *p*-coumaric acid when combined with BaPadR (S39A), while none of the engineered BsPadR systems demonstrated full induction with 600 mg/L *p*-coumaric acid.^9^ These findings highlighted the unique ligand preference and higher dynamic ranges of the engineered BaPadR systems. Overall, the versatility in the ligand profile and increased dynamic ranges provided by BaPadR-based biosensor systems hold significant potential for further advancements in biosensor-enabled synthetic biology and metabolic engineering applications.

## Methods and Materials

### Strains, Medium, and Reagents

All strains and plasmids used in this study are listed in Table S1. *E. coli* strain XLI-Blue was used for plasmid construction and enrichment. Strain *E. coli* BW25113 F′ was used for biosensor characterization and fluorescence assay. LB medium containing 10 g/L NaCl, 5 g/L yeast extract, and 10 g/L tryptone medium was utilized for the *E. coli* culture. Ampicillin and kanamycin were supplemented in the medium as needed with the final concentrations of 100 and 50 mg/mL, respectively. Different concentration of IPTG was added into medium if needed. For the inducer preparation, 100 mg of *p*-coumaric acid, caffeic acid, *trans*-cinnamic acid, ferulic acid, 4-hydeoxybenzoic acid, anthranilic acid, salicylic acid, and vanillic acid were dissolved in 1 mL of methanol to make the master stock with a concentration of 100 g/L. Ferulic acid and *p*-coumaric acid were purchased from MP Biomedicals. Caffeic acid, anthranilic acid, and cinnamic acid were purchased from Sigma-Aldrich. Salicylic acid and vanillic acid were purchased from Alfa Aesar.

### DNA Manipulation

All genetic components are listed in Table S2. The high-copy plasmid pHA-MCS was constructed in our lab, which contains a ColE1 origin, an ampicillin resistance gene, pLlacO1 promoter, and T1 terminator as reported in the previous study.^15^ The plasmid also carries a synthetic multicloning site (MCS) that sequentially contains the recognition sites of Acc65I, NdeI, BsrGI, *Sal*I, ClaI, *Hin*dIII, NheI, *Bam*HI, and MluI. pHA-egfp-MCS was constructed by inserting the coding sequence of egfp into the MCS of pHA-MCS using Acc65I and *Sal*I.

The genome of *Bacillus amyloliquefaciens* (ATCC 23350) was used as the template for cloning Ba*padR* and BaP_padC_. The BaPadR was constructed on pCS27^20^ using *Kpn*I and *Sal*I, resulting in plasmid pCS-BaPadR. The promoter BaP_padC_ was flanked by XhoI and *Kpn*I, and cloned into pHA-egfp-MCS to construct pHA-BaP_PadC_-WT-egfp. A strong engineered RBS (with a sequence of AAAGAGGAGAAA) along with an inserted *Eco*RI site was added to the BaP_padC_ promoter. The new promoter was flanked by XhoI and *Eco*RI, and was cloned into plasmid pHA-egfp-MCS to construct pHA-BaP_padC_-RBS-egfp.

Site-directed mutagenesis on residue H38 was carried out by overlap-extension PCR. The DNA fragments of the generated BaPadR variants, (BaPadR-H38A, BaPadR-H38S, BaPadR-H38D, BaPadR-H38N, BaPadR-H38E, BaPadR-H38I and BaPadR-S39A), were cloned into plasmid pCS27^20^ by *Kpn*I and *Sal*I, forming pCS-BaPadR-H38A, pCS-BaPadR-H38S, pCS-BaPadR-H38D, pCS-BaPadR-H38N, pCS-BaPadR-H38E, pCS-BaPadR-H38I and pCS-BaPadR-S39A respectively.

The truncated promoters BaP_padC_-T1, BaP_padC_-T2, BaP_padC_-T3, and BaP_padC_-T4, were amplified by using pHA-BaP_padC_-RBS-egfp as the template. The fragments were digested by XhoI and *Eco*RI and integrated into pHA-egfp-MCS to construct pHA-BaPpadC-T1-egfp, pHA-BaPpadC-T2-egfp, pHA-BaPpadC-T3-egfp, and pHA-BaPpadC-T4-egfp, respectively. The DNA fragments of hybrid promoter Phy11-egfp, Phy12-egfp, Phy21-egfp, Phy22-egfp, Phy35-egfp and Phy10-egfp were amplified using the plasmid pHA-egfp-MCS as the template. All hybrid promoter fragments were digested by XhoI and AvrII and cloned into plasmid pHA-MCS to construct pHA-Phy11-egfp, pHA-Phy12-egfp, pHA-Phy21-egfp, pHA-Phy22-egfp, pHA-Phy35-egfp, and pHA-Phy10-egfp, respectively.

Base-replacement of the BaP_padC_-RBS promoters (BaP_padC_-C6A-RBS, BaP_padC_-C6T-RBS, BaP_padC_-C6G-RBS, BaP_padC_-T8A-RBS, BaP_padC_-T8C-RBS, BaP_padC_-T8G-RBS, BaP_padC_-T18′A-RBS, BaP_padC_-T18′C-RBS, and BaP_padC_-T18′G-RBS) were amplified by using pHA-BaP_padC_-RBS-egfp as the template, and were constructed through SLIM strategy^25^ forming pHA-BaP_padC_-C6A-RBS-egfp, pHA-BaP_padC_-C6T-RBS-egfp, pHA-BaP_padC_-C6G-RBS-egfp, pHA-BaP_padC_-T8A-RBS-egfp, pHA-BaP_padC_-T8C-RBS-egfp, pHA-BaP_padC_-T8G-RBS-egfp, pHA-BaP_padC_-T18′A-RBS-egfp, pHA-BaP_padC_-T18′C-RBS-egfp, and pHA-BaP_padC_-T18′G-RBS-egfp, respectively. By using the pHA-Phy12-egfp as the template, DNA fragments containing the base-replaced promoter (Phy12-C6A-egfp, Phy12-C6T-egfp, Phy12-C6G-egfp, Phy12-T8A-egfp, Phy12-T8C-egfp, Phy12-T8G-egfp, Phy12-T18′A-egfp, Phy12-T18′C-egfp, Phy12-T18′G-egfp) were amplified. All the base-replaced Phy12 promoter fragments were digested by XhoI and *Hin*dIII, and were constructed into the pHA-MCS plasmid to construct pHA-Phy12-C6A-egfp, pHA-Phy12-C6T-egfp, pHA-Phy12-C6G-egfp, pHA-Phy12-T8A-egfp, pHA-Phy12-T8C-egfp, pHA-Phy12-T8G-egfp, pHA-Phy12-T18′A-egfp, pHA-Phy12-T18′C-egfp, and pHA-Phy12-T18′G-egfp, respectively.

### Dynamic Performance Characterization

The transformants were grown at 37 °C for overnight. Three single colonies were randomly picked and inoculated into 3.5 mL of LB medium containing specific antibiotics. After 10 h of cultivation at 37 °C, 150 μL cultures were used as seeds and were transformed into 3.5 mL of fresh LB medium containing specific antibiotics. Gradient concentrations of the ligands were added into the medium after 1 h of cultivation. Samples were collected after 12 h of cultivation and were diluted for measurement of cell densities (OD_600_) and green fluorescence intensities.

### Fluorescence Assay

40 μL of cell cultures (40 μL) were transformed into a black 96-well plate (Corning 96-well Flat Clear Bottom Black Polystyrene TC-treated Microplates, Corning 3603) and diluted with 160 μL of water. The 96-well plate carried with samples was scanned by a Synergy HT reader (BioTek). The green fluorescence intensities were detected using an excitation filter of 485/20 nm and an emission filter of 528/20 nm. The OD_600_ of cell cultures was also detected by the plate reader. The egfp expression levels were represented by normalizing the fluorescence intensities with their corresponding cell densities OD_600_. The normalized fluorescence was calculated as the equation below:
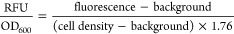
The inhibition efficiency was defined as the equation below:
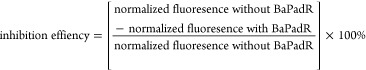


## Supporting Information Available

Sequence alignment for identifying the BaPadR and corresponding promoter BaP_padC_ (Figure S1); lists of strains, plasmids, gene sequences and promoter sequences used in this study (Tables S1–S2) (PDF)

## References

[ref1] Li C.; Jiang T.; Li M.; Zou Y.; Yan Y. Fine-tuning gene expression for improved biosynthesis of natural products: From transcriptional to post-translational regulation. Biotechnol. Adv. 2022, 54, 107853 10.1016/j.biotechadv.2021.107853. 34637919 PMC8724446

[ref2] Teng Y.; Zhang J.; Jiang T.; Zou Y.; Gong X.; Yan Y. Biosensor-enabled pathway optimization in metabolic engineering. Curr. Opin. Biotechnol. 2022, 75, 102696 10.1016/j.copbio.2022.102696. 35158314 PMC9177593

[ref3] Hossain G. S.; Saini M.; Miyake R.; Ling H.; Chang M. W. Genetic Biosensor Design for Natural Product Biosynthesis in Microorganisms. Trends Biotechnol. 2020, 38 (7), 797–810. 10.1016/j.tibtech.2020.03.013. 32359951

[ref4] Rogers J. K.; Church G. M. Genetically encoded sensors enable real-time observation of metabolite production. Proc. Natl. Acad. Sci. U.S.A. 2016, 113 (9), 2388–2393. 10.1073/pnas.1600375113. 26858408 PMC4780645

[ref5] Ding N.; Zhou S.; Deng Y. Transcription-Factor-based Biosensor Engineering for Applications in Synthetic Biology. ACS Synth. Biol. 2021, 10 (5), 911–922. 10.1021/acssynbio.0c00252. 33899477

[ref6] Mitchler M. M.; Garcia J. M.; Montero N. E.; Williams G. J. Transcription factor-based biosensors: a molecular-guided approach for natural product engineering. Curr. Opin. Biotechnol. 2021, 69, 172–181. 10.1016/j.copbio.2021.01.008. 33493842 PMC8238798

[ref7] Park S. C.; Kwak Y. M.; Song W. S.; Hong M.; Yoon S.-i. Structural basis of effector and operator recognition by the phenolic acid-responsive transcriptional regulator PadR. Nucleic Acids Res. 2017, 45 (22), 13080–13093. 10.1093/nar/gkx1055. 29136175 PMC5728393

[ref8] Nguyen T. K.; Tran N. P.; Cavin J. F. Genetic and biochemical analysis of PadR-padC promoter interactions during the phenolic acid stress response in Bacillus subtilis 168. J. Bacteriol. 2011, 193 (16), 4180–4191. 10.1128/JB.00385-11. 21685295 PMC3147689

[ref9] Jiang T.; Li C.; Yan Y. Optimization of a p-Coumaric Acid Biosensor System for Versatile Dynamic Performance. ACS Synth. Biol. 2021, 10 (1), 132–144. 10.1021/acssynbio.0c00500. 33378169 PMC8130012

[ref10] Huang Q.; Lin Y.; Yan Y. Caffeic acid production enhancement by engineering a phenylalanine over-producing Escherichia coli strain. Biotechnol. Bioeng. 2013, 110 (12), 3188–3196. 10.1002/bit.24988. 23801069

[ref11] Lin Y.; Sun X.; Yuan Q.; Yan Y. Combinatorial biosynthesis of plant-specific coumarins in bacteria. Metab. Eng. 2013, 18, 69–77. 10.1016/j.ymben.2013.04.004. 23644174

[ref12] Yang Y.; Lin Y.; Li L.; Linhardt R. J.; Yan Y. Regulating malonyl-CoA metabolism via synthetic antisense RNAs for enhanced biosynthesis of natural products. Metab. Eng. 2015, 29, 217–226. 10.1016/j.ymben.2015.03.018. 25863265

[ref13] Siedler S.; Khatri N. K.; Zsohár A.; Kjærbo̷lling I.; Vogt M.; Hammar P.; Nielsen C. F.; Marienhagen J.; Sommer M. O. A.; Joensson H. N. Development of a Bacterial Biosensor for Rapid Screening of Yeast p-Coumaric Acid Production. ACS Synth. Biol. 2017, 6 (10), 1860–1869. 10.1021/acssynbio.7b00009. 28532147

[ref14] Li C.; Zou Y.; Jiang T.; Zhang J.; Yan Y. Harnessing plasmid replication mechanism to enable dynamic control of gene copy in bacteria. Metab. Eng. 2022, 70, 67–78. 10.1016/j.ymben.2022.01.003. 35033655 PMC8844098

[ref15] Jiang T.; Li C.; Zou Y.; Zhang J.; Gan Q.; Yan Y. Establishing an Autonomous Cascaded Artificial Dynamic (AutoCAD) regulation system for improved pathway performance. Metab. Eng. 2022, 74, 1–10. 10.1016/j.ymben.2022.08.009. 36041638 PMC10947494

[ref16] Sun H.; Zhao H.; Ang E. L. A New Biosensor for Stilbenes and a Cannabinoid Enabled by Genome Mining of a Transcriptional Regulator. ACS Synth. Biol. 2020, 9 (4), 698–705. 10.1021/acssynbio.9b00443. 32078771

[ref17] Shlomi T.; Eisenberg Y.; Sharan R.; Ruppin E. A genome-scale computational study of the interplay between transcriptional regulation and metabolism. Mol. Syst. Biol. 2007, 3 (1), 101 10.1038/msb4100141. 17437026 PMC1865583

[ref18] Novichkov P. S.; Kazakov A. E.; Ravcheev D. A.; Leyn S. A.; Kovaleva G. Y.; Sutormin R. A.; Kazanov M. D.; Riehl W.; Arkin A. P.; Dubchak I.; et al. RegPrecise 3.0 – A resource for genome-scale exploration of transcriptional regulation in bacteria. BMC Genom. 2013, 14 (1), 745 10.1186/1471-2164-14-745. PMC3840689 24175918

[ref19] Waterhouse A.; Bertoni M.; Bienert S.; Studer G.; Tauriello G.; Gumienny R.; Heer F. T.; de Beer T. A P.; Rempfer C.; Bordoli L.; et al. SWISS-MODEL: homology modelling of protein structures and complexes. Nucleic Acids Res. 2018, 46 (W1), W296–W303. 10.1093/nar/gky427. 29788355 PMC6030848

[ref20] Atsumi S.; Cann A. F.; Connor M. R.; Shen C. R.; Smith K. M.; Brynildsen M. P.; Chou K. J. Y.; Hanai T.; Liao J. C. Metabolic engineering of Escherichia coli for 1-butanol production. Metab. Eng. 2008, 10 (6), 305–311. 10.1016/j.ymben.2007.08.003. 17942358

[ref21] Wang J.; Shen X.; Rey J.; Yuan Q.; Yan Y. Recent advances in microbial production of aromatic natural products and their derivatives. Appl. Microbiol. Biotechnol. 2018, 102 (1), 47–61. 10.1007/s00253-017-8599-4. 29127467

[ref22] Zhang R.; Li C.; Wang J.; Yang Y.; Yan Y. Microbial production of small medicinal molecules and biologics: From nature to synthetic pathways. Biotechnol. Adv. 2018, 36 (8), 2219–2231. 10.1016/j.biotechadv.2018.10.009. 30385278

[ref23] Lin Y.; Shen X.; Yuan Q.; Yan Y. Microbial biosynthesis of the anticoagulant precursor 4-hydroxycoumarin. Nat. Commun. 2013, 4 (1), 2603 10.1038/ncomms3603. 24129598

[ref24] Lutz R.; Bujard H. Independent and Tight Regulation of Transcriptional Units in Escherichia Coli Via the LacR/O, the TetR/O and AraC/I1-I2 Regulatory Elements. Nucleic Acids Res. 1997, 25 (6), 1203–1210. 10.1093/nar/25.6.1203. 9092630 PMC146584

[ref25] Chiu J.; March P. E.; Lee R.; Tillett D. Site-directed, Ligase-Independent Mutagenesis (SLIM): a single-tube methodology approaching 100% efficiency in 4 h. Nucleic Acids Res. 2004, 32 (21), e174–e174. 10.1093/nar/gnh172. 15585660 PMC535700

