# Supplementary material for: Identifying, Characterizing, and Engineering a Phenolic Acid-Responsive Transcriptional Factor from Bacillus amyloliquefaciens
Source: ACS Synth Biol. 2023 Jul 27;12(8):2382–92. doi: 10.1021/acssynbio.3c00206 (PMC10443031; doi:10.1021/acssynbio.3c00206)
Supplement: Supplementary file 1 — sb3c00206_si_001.pdf [file sb3c00206_si_001.pdf]

Supporting Information

Identifying, Characterizing, and Engineering a Phenolic Acid-Responsive Transcriptional Factor from *Bacillus Amyloliquefaciens*

Chenyi Li[1a], Yuyang Zhou[1a], Yusong Zou[1], Tian Jiang[1], Xinyu Gong[1], Yajun Yan[1*]

[1] School of Chemical, Materials and Biomedical Engineering, College of Engineering, The University of Georgia, Athens, GA, 30602, USA

[a] These authors contributed equally to this work

*Correspondence: yajunyan@uga.edu

(a)

```
# Aligned_sequences: 2
# 1: BsPadR
# 2: BaPadR
# Matrix: EBLOSUM62
# Gap_penalty: 10.0
# Extend_penalty: 0.5
#
# Length: 183
# Identity:     145/183 (79.2%)
# Similarity:   161/183 (88.0%)
# Gaps:           1/183 ( 0.5%)
# Score: 743.0
#
#
#=======================================

BsPadR            1 MRVLKYAILGLLRKGELSGYDITSYFKEELGQFWSAKHSQIYPELKKLTD     50
                    ||:||||||||||||||||||||:||||||||||||||||||||||||||.
BaPadR            1 MRILKYAILGLLRKGELSGYDISSYFKEELGQFWSAKHSQIYPELKKLTA     50

BsPadR           51 EGFITFRTTIQGTKLEKKMYTLTDSGKQELHDWLIRHQPIPETVKDEFML    100
                    ||||||||.||||||||||||||||:|::||..||.:..||||||||||||
BaPadR           51 EGFITFRTAIQGTKLEKKMYTLTDNGERELCAWLTKKDPIPETVKDEFML    100

BsPadR          101 KAYFISCLSRQEASDLFKDQLQKRQAKLSDLQGSYEKLMASAEPM-SFSS    149
                    ||||||.|:.:||.:||.|||.||:.|||||:.||.:||.|:|.. ||||
BaPadR          101 KAYFISALTNEEADELFTDQLVKRKEKLSDLENSYHELMTSSEEADSFSS    150

BsPadR          150 PDFGHYLVLTKALEREKNYVSWLESILAMIDKD    182
                    ||||||||||||||||:||:||||.|||:|.|.
BaPadR          151 PDFGHYLVLTKALERERNYISWLEHILALIKKA    183
```

(b)

```
# Aligned_sequences: 2
# 1: BsPadC
# 2: BaPadC
# Matrix: EBLOSUM62
# Gap_penalty: 10.0
# Extend_penalty: 0.5
#
# Length: 161
# Identity:     147/161 (91.3%)
# Similarity:   157/161 (97.5%)
# Gaps:           0/161 ( 0.0%)
# Score: 827.0
#
#
#=======================================

BsPadC            1 MENFIGSHMIYTYENGWEYEIYIKNDHTIDYRIHSGMVAGRWVRDQEVNI     50
                    ||||||||||||||||||||||||||||||||||||||.|||||||||||
BaPadC            1 MENFIGSHMIYTYENGWEYEIYIKNDHTIDYRIHSGMVGGRWVRDQEVNI     50

BsPadC           51 VKLTEGVYKVSWTEPTGTDVSLNFMPNEKRMHGIIFFPKWVHEHPEITVC    100
                    ||||||||||||||||||||||||||||||||||||||||||||||||||
BaPadC           51 VKLTEGVYKVSWTEPTGTDVSLNFMPNEKRMHGIIFFPKWVHEHPEITVC    100

BsPadC          101 YQNDHIDLMKESREKYETYPKYVVPEFAEITFLKNEGVDNEEVISKAPYE    150
                    ||||:||:||||||||:|||||||||||:||:|.|.|::||.:||:||||
BaPadC          101 YQNDYIDVMKESREKYDTYPKYVVPEFADITYLNNAGINNEALISEAPYE    150

BsPadC          151 GMTDDIRAGRL    161
                    |||||||||:|
BaPadC          151 GMTDDIRAGKL    161
```

(c) BaP$_{padC}$ promoter

GATTCCTTTCTAGTTCGCGAGCCGCATGTGAGCATGGACCTAAGGAGCGCCAGAGTAAATGAAAAAGACAAGGGTTTCGGCATTTGCCGTCCTTGTCTTTTTTATTAAACA

BaPadR-1

CCTTTTTACCGGGTGAAGCATCTCATCATTTGACAGTCCATTTTAACAGCGTTACAATTAATCATGTAAATAGTTACATGTATATATAAACATAATGTCTTGGGAGGTGATAC

BaPadR-2

BayveG ?

GTCCTGAATGAACCTTCTTTAAAAAAAGTGATGTCTCTGCAAATTAAAGTGGATGAACCGATCGTTGCTGGGCAGACTGGATTAGGGAAAAGGCAGCTGATCCTGATTCGG

GGCGGAACGGTCAGCGGCGCCGTAAAGGGGCGTGTTCTCCCGGGCGGGGCAGACGCCCAGATCATCCGTCCGAACGGCCGGGTTGATCTTTCTGCTAGGTACGCGCTG

GAAACCGAAGAGCACGAAGTCATTTATGTTGAGAACAACGGCATCCGCCAAGTCAGTGAACCGTTCCGCCAGCAGGCTGCAGAGGGGCGCATCATTGATCACGAGCACG

TGTATTTCCGCACTGTGCCTGTGTTTAAAACAAGCAGTAAGGCATATCAGCATCTGCAGGACCGGATGTTTATCGGGGCTGCGGTCAGATTGCCGGATGACATTCGGTTGG

BapadC

ATATTTATGAAGTTCAATAATAATAATATGGAGAGTGAGTTAGCATG

(d)

```
# Aligned_sequences: 2
# 1: BsYveG
# 2: BaYveG-core
# Matrix: EBLOSUM62
# Gap_penalty: 10.0
# Extend_penalty: 0.5
#
# Length: 142
# Identity:      66/142 (46.5%)
# Similarity:    79/142 (55.6%)
# Gaps:          47/142 (33.1%)
# Score: 352.0
#
#
#=======================================

BsYveG            1 -----------------------------------------------MIR      3
                                                                   :||
BaYveG            1 MSLQIKVDEPIVAGQTGLGKRQLILIRGGTVSGAVKGRVLPGGADAQIIR     50

BsYveG            4 ANGRTDLSARYVIETADHELIYIENNGIRQVSKPFRKQAAAGEIIEPEHV     53
                    .|||.||||||.:||.:||:||:|||||||||:|||:|||.|.||:.|||
BaYveG           51 PNGRVDLSARYALETEEHEVIYVENNGIRQVSEPFRQQAAEGRIIDHEHV    100

BsYveG           54 YFRTVPTFETGSEVYQWLHDRLFIGSAERTPDYVLLDIYEVQ     95
                    ||||||.|:|.|:.||.|.||:|||:|.|.||.:.|||||||
BaYveG          101 YFRTVPVFKTSSKAYQHLQDRMFIGAAVRLPDDIRLDIYEVQ    142
```

(e)

```
# Aligned_sequences: 2
# 1: BsYveG
# 2: BaYveG-core
# Matrix: EBLOSUM62
# Gap_penalty: 10.0
# Extend_penalty: 0.5
#
# Length: 96
# Identity:      66/96 (68.8%)
# Similarity:    79/96 (82.3%)
# Gaps:           1/96 ( 1.0%)
# Score: 352.0
#
#
#=======================================

BsYveG            1 -MIRANGRTDLSARYVIETADHELIYIENNGIRQVSKPFRKQAAAGEIIE     49
                     :||.|||.||||||.:||.:||:||:|||||||||:|||:|||.|.||:
BaYveG-core       1 QIIRPNGRVDLSARYALETEEHEVIYVENNGIRQVSEPFRQQAAEGRIID     50

BsYveG           50 PEHVYFRTVPTFETGSEVYQWLHDRLFIGSAERTPDYVLLDIYEVQ     95
                    .||||||||||.|:|.|:.||.|.||:|||:|.|.||.:.|||||||
BaYveG-core      51 HEHVYFRTVPVFKTSSKAYQHLQDRMFIGAAVRLPDDIRLDIYEVQ     96
```

Supplementary Figure S1. Protein sequence alignment. (a) Protein sequence alignment of BaPadR and BsPadR. The red rectangle (rounded corners) highlighted the two regions that showed most variations in sequence between these two proteins. **(b)** Protein sequence alignment of BaPadC and BsPadC. **(c)** The upstream DNA sequence of Ba*padC* gene. Green sequence is used as the BaP_{padC} promoter in this study. Blue sequence is the suspected BaYveG encoding gene which is homologous to BsYveG. The Yellow sequence with a underline is the suspected BaPadR binding box. **(d)** Protein sequence alignment of BaYveG and BsYveG. The red rectangle (rounded corners) highlighted a redundant sequence in the N-terminus of BaYveG that did not have any match against BsYveG. **(e)** Protein sequence alignment of the truncated BaYveG and BsYveG.

Supplementary Table S1. Strains and Plasmids Used in This Study

Name	Genotype	Reference
E. coli XLI-Blue	*recA1 endA1gyrA96thi-1hsdR17supE44relA1lac [F' proAB lacIqZDM15Tn10 (TetR)]*	Stratagene
E. coli BW25113 F'	*rrnBT14 ΔlacZWJ16 hsdR514 ΔaraBADAH33 ΔrhaBADLD78 F' [traD36 proAB lacIqZΔM15 Tn10(Tetr)]*	[1]
**Plasmid**	**Description**	**Reference**
pCS27	pL_{lacO1}; *p15A ori*; *Kan*^{R}	[1]
pHA-MCS	pL_{lacO1}; *ColE1 ori*, *Amp*^{R}	[2]
pHA-egfp-MCS	pHA-MCS containing pLlacO1-controlled *egfp* expression cassette	This study
pHA-BaP_{padC}-WT-egfp	pHA-egfp-MCS harboring the wild type PadC promoter (BaP_{padC}) and RBS region from *Bacillus amyloliquefaciens*	This study
pHA-BaP_{padC}-RBS-egfp	pHA-egfp-MCS harboring the wild type PadC promoter from *Bacillus amyloliquefaciens* with the strong engineered RBS inserted between the BaP_{padC} promoter and the *egfp* gene	This study
pCS-BaPadR	pCS27 with a pL_{lacO1}-controlled wild type BaPadR expression cassette	This study
pCS-BaPadR-H38A	pCS27 carrying BaPadR mutant H38A	This study

pCS-BaPadR-H38S	pCS27 carrying PadR mutant H38S	This study
pCS-BaPadR-H38D	pCS27 carrying PadR mutant H38D	This study
pCS-BaPadR-H38N	pCS27 carrying PadR mutant H38N	This study
pCS-BaPadR-H38E	pCS27 carrying PadR mutant H38E	This study
pCS-BaPadR-H38I	pCS27 carrying PadR mutant H38I	This study
pCS-BaPadR-S39A	pCS27 carrying PadR mutant S39A	This study
pHA-BaP$_{padC}$-T1-egfp	pHA-egfp-MCS carrying the truncated BaP$_{padC}$ promoter T1 and the strong RBS region	This study
pHA-BaP$_{padC}$-T2-egfp	pHA-egfp-MCS carrying the truncated BaP$_{padC}$ promoter T2 and the strong RBS region	This study
pHA-BaP$_{padC}$-T3-egfp	pHA-egfp-MCS carrying the truncated BaP$_{padC}$ promoter T3 and the strong RBS region	This study
pHA-BaP$_{padC}$-T4-egfp	pHA-egfp-MCS carrying the truncated BaP$_{padC}$ promoter T4 and the strong RBS region	This study
pHA- Phy11-egfp	pHA-egfp-MCS harboring the hybrid promoter Phy11	This study
pHA- Phy12-egfp	pHA-egfp-MCS harboring the hybrid promoter Phy12	This study
pHA- Phy21-egfp	pHA-egfp-MCS harboring the hybrid promoter Phy21	This study
pHA- Phy22-egfp	pHA-egfp-MCS harboring the hybrid promoter Phy22	This study
pHA- Phy10-egfp	pHA-egfp-MCS harboring the hybrid promoter Phy10	This study
pHA- Phy35-egfp	pHA-egfp-MCS harboring the hybrid promoter Phy35	This study

pHA-BaPpadC-C6A-RBS-egfp	pHA-egfp-MCS harboring the BaP$_{padC}$-C6A promoter variant with the strong engineered RBS inserted between the BaP$_{padC}$ promoter and the *egfp* gene	This study
pHA-BaPpadC-C6T-RBS-egfp	pHA-egfp-MCS harboring the BaP$_{padC}$-C6T promoter variant with the strong engineered RBS inserted between the BaP$_{padC}$ promoter and the *egfp* gene	This study
pHA-BaPpadC-C6G-RBS-egfp	pHA-egfp-MCS harboring the BaP$_{padC}$-C6G promoter variant with the strong engineered RBS inserted between the BaP$_{padC}$ promoter and the *egfp* gene	This study
pHA-BaPpadC-T8A-RBS-egfp	pHA-egfp-MCS harboring the BaP$_{padC}$-T8A promoter variant with the strong engineered RBS inserted between the BaP$_{padC}$ promoter and the *egfp* gene	This study
pHA-BaPpadC-T8C-RBS-egfp	pHA-egfp-MCS harboring the BaP$_{padC}$-T8C promoter variant with the strong engineered RBS inserted between the BaPpadC promoter and the egfp gene	This study
pHA-BaPpadC-T8G-RBS-egfp	pHA-egfp-MCS harboring the BaPpadC-T8G promoter variant with the strong engineered RBS inserted between the BaPpadC promoter and the egfp gene	This study
pHA-BaPpadC-T18’A-RBS-egfp	pHA-egfp-MCS harboring the BaP$_{padC}$-T18’A promoter variant with the strong engineered RBS inserted between the BaPpadC promoter and the egfp gene	This study

pHA-BaPpadC-T18’C-RBS-egfp	pHA-egfp-MCS harboring the BaPpadC-T18’C promoter variant with the strong engineered RBS inserted between the BaPpadC promoter and the egfp gene	This study
pHA-BaPpadC-T18’G-RBS-egfp	pHA-egfp-MCS harboring the BaPpadC-T18’G promoter variant with the strong engineered RBS inserted between the BaPpadC promoter and the egfp gene	This study
pHA-Phy21-C6A-egfp	pHA-egfp-MCS harboring the hybrid promoter Phy12-C6A	This study
pHA-Phy21-C6T-egfp	pHA-egfp-MCS harboring the hybrid promoter Phy12-C6T	This study
pHA-Phy21-C6G-egfp	pHA-egfp-MCS harboring the hybrid promoter Phy12-C6G	This study
pHA-Phy21-T8A-egfp	pHA-egfp-MCS harboring the hybrid promoter Phy12-T8A	This study
pHA-Phy21-T8C-egfp	pHA-egfp-MCS harboring the hybrid promoter Phy12-T8C	This study
pHA-Phy21-T8G-egfp	pHA-egfp-MCS harboring the hybrid promoter Phy12-T8G	This study
pHA-Phy21-T18’A-egfp	pHA-egfp-MCS harboring the hybrid promoter Phy12-T18’A	This study
pHA-Phy21-T18’C-egfp	pHA-egfp-MCS harboring the hybrid promoter Phy12-T18’C	This study

pHA-Phy21-T18’G-egfp	pHA-egfp-MCS harboring the hybrid promoter Phy12-T18’G	This study

Supplementary Table S2 The DNA sequence of all components used in this study

Name	DNA sequence (5'-3') [a]
BaP$_{padC}$-WT	GATTCCTTTCTAGTTCGCGAGCCGCATGTGAGCATGGACCTAAGGAGCGCCAGAGTAAA<br>TGAAAAAGACAAGGGTTTCGGCATTTGCCGTCCTTGTCTTTTTTATTAAACACCTTTTTAC<br>CGGGTGAAGCATCTCATCATTTGACAGTCCATTTTAACAGCGTTACAATTAATCATGTAA<br>ATAGTTACATGTATATATAAACATAATGTCTTGGGAGGTGATACGTCCTGAATGAACCTT<br>CTTTAAAAAAAGTG
BaP$_{padC}$-RBS [b]	GATTCCTTTCTAGTTCGCGAGCCGCATGTGAGCATGGACCTAAGGAGCGCCAGAGTAAA<br>TGAAAAAGACAAGGGTTTCGGCATTTGCCGTCCTTGTCTTTTTTATTAAACACCTTTTTAC<br>CGGGTGAAGCATCTCATCATTTGACAGTCCATTTTAACAGCGTTACAATTAATCATGTAA<br>ATAGTTACATGTATATATAAACATAATGTCTTGGGAGGTGATACGTCCTGAATGAACCTT<br>CTTTAAAAAAAGTGGAATTCATT**AAAGAGGAGAAA**
BaPadR	ATGAGAATTTTAAAGTACGCGATTTTAGGACTTTTGCGAAAAGGCGAATTGAGCGGATA<br>TGATATATCGAGCTATTTTAAAGAAGAGCTAGGCCAGTTTTGGAGCGCAAAGCACAGCC<br>AGATTTATCCGGAATTAAAAAAGCTGACGGCTGAGGGATTCATTACGTTCCGCACTGCG<br>ATTCAGGGAACGAAGCTGGAGAAAAAAATGTACACGCTGACTGACAATGGAGAGCGGG<br>AGCTTTGTGCATGGCTGACGAAAAAAGATCCGATTCCGGAAACGGTGAAGGATGAATTT<br>ATGCTGAAGGCTTATTTTATCTCAGCTTTGACGAATGAAGAAGCGGATGAGCTATTCACC<br>GATCAGCTCGTAAAGCGAAAGGAGAAGTTGTCCGATCTGGAAAACAGTTATCATGAACT<br>GATGACATCCTCCGAGGAGGCGGATTCCTTTTCTTCTCCGGATTTCGGCCATTATCTCGT<br>GCTGACAAAAGCGCTGGAGCGGGAAAGGAATTATATTTCCTGGCTTGAGCATATTTTGG<br>CTCTCATCAAAAAAGCATAA
BaPadR-H38A	ATGAGAATTTTAAAGTACGCGATTTTAGGACTTTTGCGAAAAGGCGAATTGAGCGGATA<br>TGATATATCGAGCTATTTTAAAGAAGAGCTAGGCCAGTTTTGGAGCGCAAAGGCGAGCC<br>AGATTTATCCGGAATTAAAAAAGCTGACGGCTGAGGGATTCATTACGTTCCGCACTGCG<br>ATTCAGGGAACGAAGCTGGAGAAAAAAATGTACACGCTGACTGACAATGGAGAGCGGG<br>AGCTTTGTGCATGGCTGACGAAAAAAGATCCGATTCCGGAAACGGTGAAGGATGAATTT<br>ATGCTGAAGGCTTATTTTATCTCAGCTTTGACGAATGAAGAAGCGGATGAGCTATTCACC<br>GATCAGCTCGTAAAGCGAAAGGAGAAGTTGTCCGATCTGGAAAACAGTTATCATGAACT<br>GATGACATCCTCCGAGGAGGCGGATTCCTTTTCTTCTCCGGATTTCGGCCATTATCTCGT

GCTGACAAAAGCGCTGGAGCGGGAAAGGAATTATATTTCCTGGCTTGAGCATATTTTGG
CTCTCATCAAAAAAGCATAA

BaPadR-H38S	ATGAGAATTTTAAAGTACGCGATTTTAGGACTTTTGCGAAAAGGCGAATTGAGCGGATA TGATATATCGAGCTATTTTAAAGAAGAGCTAGGCCAGTTTTGGAGCGCAAAGAGCAGCC AGATTTATCCGGAATTAAAAAAGCTGACGGCTGAGGGATTCATTACGTTCCGCACTGCG ATTCAGGGAACGAAGCTGGAGAAAAAAATGTACACGCTGACTGACAATGGAGAGCGGG AGCTTTGTGCATGGCTGACGAAAAAAGATCCGATTCCGGAAACGGTGAAGGATGAATTT ATGCTGAAGGCTTATTTTATCTCAGCTTTGACGAATGAAGAAGCGGATGAGCTATTCACC GATCAGCTCGTAAAGCGAAAGGAGAAGTTGTCCGATCTGGAAAACAGTTATCATGAACT GATGACATCCTCCGAGGAGGCGGATTCCTTTTCTTCTCCGGATTTCGGCCATTATCTCGT GCTGACAAAAGCGCTGGAGCGGGAAAGGAATTATATTTCCTGGCTTGAGCATATTTTGG CTCTCATCAAAAAAGCATAA
BaPadR-H38D	ATGAGAATTTTAAAGTACGCGATTTTAGGACTTTTGCGAAAAGGCGAATTGAGCGGATA TGATATATCGAGCTATTTTAAAGAAGAGCTAGGCCAGTTTTGGAGCGCAAAGGATAGCC AGATTTATCCGGAATTAAAAAAGCTGACGGCTGAGGGATTCATTACGTTCCGCACTGCG ATTCAGGGAACGAAGCTGGAGAAAAAAATGTACACGCTGACTGACAATGGAGAGCGGG AGCTTTGTGCATGGCTGACGAAAAAAGATCCGATTCCGGAAACGGTGAAGGATGAATTT ATGCTGAAGGCTTATTTTATCTCAGCTTTGACGAATGAAGAAGCGGATGAGCTATTCACC GATCAGCTCGTAAAGCGAAAGGAGAAGTTGTCCGATCTGGAAAACAGTTATCATGAACT GATGACATCCTCCGAGGAGGCGGATTCCTTTTCTTCTCCGGATTTCGGCCATTATCTCGT GCTGACAAAAGCGCTGGAGCGGGAAAGGAATTATATTTCCTGGCTTGAGCATATTTTGG CTCTCATCAAAAAAGCATAA
BaPadR-H38N	ATGAGAATTTTAAAGTACGCGATTTTAGGACTTTTGCGAAAAGGCGAATTGAGCGGATA TGATATATCGAGCTATTTTAAAGAAGAGCTAGGCCAGTTTTGGAGCGCAAAGAACAGCC AGATTTATCCGGAATTAAAAAAGCTGACGGCTGAGGGATTCATTACGTTCCGCACTGCG ATTCAGGGAACGAAGCTGGAGAAAAAAATGTACACGCTGACTGACAATGGAGAGCGGG AGCTTTGTGCATGGCTGACGAAAAAAGATCCGATTCCGGAAACGGTGAAGGATGAATTT ATGCTGAAGGCTTATTTTATCTCAGCTTTGACGAATGAAGAAGCGGATGAGCTATTCACC GATCAGCTCGTAAAGCGAAAGGAGAAGTTGTCCGATCTGGAAAACAGTTATCATGAACT GATGACATCCTCCGAGGAGGCGGATTCCTTTTCTTCTCCGGATTTCGGCCATTATCTCGT

	GCTGACAAAAGCGCTGGAGCGGGAAAGGAATTATATTTCCTGGCTTGAGCATATTTTGG<br>CTCTCATCAAAAAAGCATAA
BaPadR-H38E	ATGAGAATTTTAAAGTACGCGATTTTAGGACTTTTGCGAAAAGGCGAATTGAGCGGATA<br>TGATATATCGAGCTATTTTAAAGAAGAGCTAGGCCAGTTTTGGAGCGCAAAGGAAAGCC<br>AGATTTATCCGGAATTAAAAAAGCTGACGGCTGAGGGATTCATTACGTTCCGCACTGCG<br>ATTCAGGGAACGAAGCTGGAGAAAAAAATGTACACGCTGACTGACAATGGAGAGCGGG<br>AGCTTTGTGCATGGCTGACGAAAAAAGATCCGATTCCGGAAACGGTGAAGGATGAATTT<br>ATGCTGAAGGCTTATTTTATCTCAGCTTTGACGAATGAAGAAGCGGATGAGCTATTCACC<br>GATCAGCTCGTAAAGCGAAAGGAGAAGTTGTCCGATCTGGAAAACAGTTATCATGAACT<br>GATGACATCCTCCGAGGAGGCGGATTCCTTTTCTTCTCCGGATTTCGGCCATTATCTCGT<br>GCTGACAAAAGCGCTGGAGCGGGAAAGGAATTATATTTCCTGGCTTGAGCATATTTTGG<br>CTCTCATCAAAAAAGCATAA
BaPadR-H38I	ATGAGAATTTTAAAGTACGCGATTTTAGGACTTTTGCGAAAAGGCGAATTGAGCGGATA<br>TGATATATCGAGCTATTTTAAAGAAGAGCTAGGCCAGTTTTGGAGCGCAAAGATTAGCC<br>AGATTTATCCGGAATTAAAAAAGCTGACGGCTGAGGGATTCATTACGTTCCGCACTGCG<br>ATTCAGGGAACGAAGCTGGAGAAAAAAATGTACACGCTGACTGACAATGGAGAGCGGG<br>AGCTTTGTGCATGGCTGACGAAAAAAGATCCGATTCCGGAAACGGTGAAGGATGAATTT<br>ATGCTGAAGGCTTATTTTATCTCAGCTTTGACGAATGAAGAAGCGGATGAGCTATTCACC<br>GATCAGCTCGTAAAGCGAAAGGAGAAGTTGTCCGATCTGGAAAACAGTTATCATGAACT<br>GATGACATCCTCCGAGGAGGCGGATTCCTTTTCTTCTCCGGATTTCGGCCATTATCTCGT<br>GCTGACAAAAGCGCTGGAGCGGGAAAGGAATTATATTTCCTGGCTTGAGCATATTTTGG<br>CTCTCATCAAAAAAGCATAA
BaPadR-S39A	ATGAGAATTTTAAAGTACGCGATTTTAGGACTTTTGCGAAAAGGCGAATTGAGCGGATA<br>TGATATATCGAGCTATTTTAAAGAAGAGCTAGGCCAGTTTTGGAGCGCAAAGCACGCGC<br>AGATTTATCCGGAATTAAAAAAGCTGACGGCTGAGGGATTCATTACGTTCCGCACTGCG<br>ATTCAGGGAACGAAGCTGGAGAAAAAAATGTACACGCTGACTGACAATGGAGAGCGGG<br>AGCTTTGTGCATGGCTGACGAAAAAAGATCCGATTCCGGAAACGGTGAAGGATGAATTT<br>ATGCTGAAGGCTTATTTTATCTCAGCTTTGACGAATGAAGAAGCGGATGAGCTATTCACC<br>GATCAGCTCGTAAAGCGAAAGGAGAAGTTGTCCGATCTGGAAAACAGTTATCATGAACT<br>GATGACATCCTCCGAGGAGGCGGATTCCTTTTCTTCTCCGGATTTCGGCCATTATCTCGT

	GCTGACAAAAGCGCTGGAGCGGGAAAGGAATTATATTTCCTGGCTTGAGCATATTTTGGCTCTCATCAAAAAAGCATAA
P_{PadC}-RBS-T1	GATTCCTTTCTAGTTCGCGAGCCGCATGTGAGCATGGACCTAAGGAGCGCCAGAGTAAATGAAAAAGACAAGGGTTTCGGCATTTGCCGTCCTTGTCTTTTTTATTAAACACCTTTTTACCGGGTGAAGCATCTCATCATTTGACAGTCCATTTTAACAGCGTTACAATTAATCATGTAAATAGTTACATGTATATATAAACATAATGTCTTGGGAGGTGATAC
P_{PadC}-RBS-T2	GATTCCTTTCTAGTTCGCGAGCCGCATGTGAGCATGGACCTAAGGAGCGCCAGAGTAAATGAAAAAGACAAGGGTTTCGGCATTTGCCGTCCTTGTCTTTTTTATTAAACACCTTTTTACCGGGTGAAGCATCTCATCATTTGACAGTCCATTTTAACAGCGTTACAATTAATCATGTAAATAGTTACATGTAT
P_{PadC}-RBS-T3	GATTCCTTTCTAGTTCGCGAGCCGCATGTGAGCATGGACCTAAGGAGCGCCAGAGTAAATGAAAAAGACAAGGGTTTCGGCATTTGCCGTCCTTGTCTTTTTTATTAAACACCTTTTTACCGGGTGAAGCATCTCATCATTTGACAGTCCATTTTAACAGCGTT
P_{PadC}-RBS-T4	GATTCCTTTCTAGTTCGCGAGCCGCATGTGAGCATGGACCTAAGGAGCGCCAGAGTAAATGAAAAAGACAAGGGTTTCGGCATTTGCCGTCCTTGTCTTTTTTATTAAACACCTTTTTACCGGGTGAAGCATCT
Original BaPadR binding sequence	CATGTAAATAGTTACATGTATATATAAACATA
BaPadR-1	CATGTAAATAGTTACATG
BaPadR-2	CATGTAAATAGTTACATG
Phy11[c]	ACATGTAAATAGTTACATGTTGACACATGTAAATAGTTACATGATACTGAGCACATCAGCAGGACGCACTGACC
Phy12[c]	ACATGTAAATAGTTACATGTTGACACATGTATATATAAACATAATACTGAGCACATCAGCAGGACGCACTGACC
Phy21[c]	ACATGTATATATAAACATATTGACACATGTAAATAGTTACATGATACTGAGCACATCAGCAGGACGCACTGACC
Phy22[c]	ACATGTATATATAAACATATTGACACATGTATATATAAACATAATACTGAGCACATCAGCAGGACGCACTGACC
Phy35[d]	TAAATTCATGTAAATAGTTTTGACAATATATAAACATAACAAGATACTGAGCACATCAGCAGGACGCACTGACC

Phy10[d]	TAAATTATCTCTGGCGGTGTTGACATCATGTAAATAGTTACAGATACTTATAAACATAGC AGGACGCACTGACC
P_{PadC}-C6A-RBS[d,e]	GATTCCTTTCTAGTTCGCGAGCCGCATGTGAGCATGGACCTAAGGAGCGCCAGAGTAAA TGAAAAAGACAAGGGTTTCGGCATTTGCCGTCCTTGTCTTTTTTATTAAACACCTTTTTAC CGGGTGAAGCATCTCATCATTTGACAGTCCATTTTAACAGCGTTACAATTAATAATGTAA ATAGTTACATGTATATATAAACATAATGTCTTGGGAGGTGATACGTCCTGAATGAACCTT CTTTAAAAAAAGTGGAATTCATTAAAGAGGAGAAA
P_{padC} -C6T-RBS[d,e]	GATTCCTTTCTAGTTCGCGAGCCGCATGTGAGCATGGACCTAAGGAGCGCCAGAGTAAA TGAAAAAGACAAGGGTTTCGGCATTTGCCGTCCTTGTCTTTTTTATTAAACACCTTTTTAC CGGGTGAAGCATCTCATCATTTGACAGTCCATTTTAACAGCGTTACAATTAATTATGTAA ATAGTTACATGTATATATAAACATAATGTCTTGGGAGGTGATACGTCCTGAATGAACCTT CTTTAAAAAAAGTGGAATTCATTAAAGAGGAGAAA
P_{padC} -C6G-RBS[d,e]	GATTCCTTTCTAGTTCGCGAGCCGCATGTGAGCATGGACCTAAGGAGCGCCAGAGTAAA TGAAAAAGACAAGGGTTTCGGCATTTGCCGTCCTTGTCTTTTTTATTAAACACCTTTTTAC CGGGTGAAGCATCTCATCATTTGACAGTCCATTTTAACAGCGTTACAATTAATGATGTAA ATAGTTACATGTATATATAAACATAATGTCTTGGGAGGTGATACGTCCTGAATGAACCTT CTTTAAAAAAAGTGGAATTCATTAAAGAGGAGAAA
P_{padC} -T8A-RBS[d,e]	GATTCCTTTCTAGTTCGCGAGCCGCATGTGAGCATGGACCTAAGGAGCGCCAGAGTAAA TGAAAAAGACAAGGGTTTCGGCATTTGCCGTCCTTGTCTTTTTTATTAAACACCTTTTTAC CGGGTGAAGCATCTCATCATTTGACAGTCCATTTTAACAGCGTTACAATTAATCAAGTAA ATAGTTACATGTATATATAAACATAATGTCTTGGGAGGTGATACGTCCTGAATGAACCTT CTTTAAAAAAAGTGGAATTCATTAAAGAGGAGAAA
P_{padC} -T8C-RBS[d,e]	GATTCCTTTCTAGTTCGCGAGCCGCATGTGAGCATGGACCTAAGGAGCGCCAGAGTAAA TGAAAAAGACAAGGGTTTCGGCATTTGCCGTCCTTGTCTTTTTTATTAAACACCTTTTTAC CGGGTGAAGCATCTCATCATTTGACAGTCCATTTTAACAGCGTTACAATTAATCACGTAA ATAGTTACATGTATATATAAACATAATGTCTTGGGAGGTGATACGTCCTGAATGAACCTT CTTTAAAAAAAGTGGAATTCATTAAAGAGGAGAAA
P_{padC} -T8G-RBS[d,e]	GATTCCTTTCTAGTTCGCGAGCCGCATGTGAGCATGGACCTAAGGAGCGCCAGAGTAAA TGAAAAAGACAAGGGTTTCGGCATTTGCCGTCCTTGTCTTTTTTATTAAACACCTTTTTAC CGGGTGAAGCATCTCATCATTTGACAGTCCATTTTAACAGCGTTACAATTAATCAGGTAA

	ATAGTTACATGTATATATAAACATAATGTCTTGGGAGGTGATACGTCCTGAATGAACCTT CTTTAAAAAAAGTGGAATTCATTAAAGAGGAGAAA
P_{padC} -T18'A-RBS[d,f]	GATTCCTTTCTAGTTCGCGAGCCGCATGTGAGCATGGACCTAAGGAGCGCCAGAGTAAA TGAAAAAGACAAGGGTTTCGGCATTTGCCGTCCTTGTCTTTTTTATTAAACACCTTTTTAC CGGGTGAAGCATCTCATCATTTGACAGTCCATTTTAACAGCGTTACAATTAATCATGTTA ATAGTTACATGTATATATAAACATAATGTCTTGGGAGGTGATACGTCCTGAATGAACCTT CTTTAAAAAAAGTGGAATTCATTAAAGAGGAGAAA
P_{padC} -T18'C-RBS[d,f]	GATTCCTTTCTAGTTCGCGAGCCGCATGTGAGCATGGACCTAAGGAGCGCCAGAGTAAA TGAAAAAGACAAGGGTTTCGGCATTTGCCGTCCTTGTCTTTTTTATTAAACACCTTTTTAC CGGGTGAAGCATCTCATCATTTGACAGTCCATTTTAACAGCGTTACAATTAATCATGTGA ATAGTTACATGTATATATAAACATAATGTCTTGGGAGGTGATACGTCCTGAATGAACCTT CTTTAAAAAAAGTGGAATTCATTAAAGAGGAGAAA
P_{padC} -T18'G-RBS[d,f]	GATTCCTTTCTAGTTCGCGAGCCGCATGTGAGCATGGACCTAAGGAGCGCCAGAGTAAA TGAAAAAGACAAGGGTTTCGGCATTTGCCGTCCTTGTCTTTTTTATTAAACACCTTTTTAC CGGGTGAAGCATCTCATCATTTGACAGTCCATTTTAACAGCGTTACAATTAATCATGTCA ATAGTTACATGTATATATAAACATAATGTCTTGGGAGGTGATACGTCCTGAATGAACCTT CTTTAAAAAAAGTGGAATTCATTAAAGAGGAGAAA
Phy12-C6A[c, e]	AAATGTAAATAGTTACATGTTGACACATGTATATATAAACATAATACTGAGCACATCAG CAGGACGCACTGACC
Phy12-C6T[c, e]	ATATGTAAATAGTTACATGTTGACACATGTATATATAAACATAATACTGAGCACATCAG CAGGACGCACTGACC
Phy12-C6G[c, e]	AGATGTAAATAGTTACATGTTGACACATGTATATATAAACATAATACTGAGCACATCAG CAGGACGCACTGACC
Phy12-T8A[c, e]	ACAAGTAAATAGTTACATGTTGACACATGTATATATAAACATAATACTGAGCACATCAG CAGGACGCACTGACC
Phy12-T8C[c, e]	ACACGTAAATAGTTACATGTTGACACATGTATATATAAACATAATACTGAGCACATCAG CAGGACGCACTGACC
Phy12-T8G[c, e]	ACAGGTAAATAGTTACATGTTGACACATGTATATATAAACATAATACTGAGCACATCAG CAGGACGCACTGACC
Phy12-T18'A[c, f]	ACATGTTAATAGTTACATGTTGACACATGTATATATAAACATAATACTGAGCACATCAG CAGGACGCACTGACC

Phy12-T18'C[c, f]	ACATGTGAATAGTTACATGTTGACACATGTATATATAAACATAATACTGAGCACATCAG CAGGACGCACTGACC
Phy12-T18'G[c, f]	ACATGTCAATAGTTACATGTTGACACATGTATATATAAACATAATACTGAGCACATCAG CAGGACGCACTGACC

[a] The mutations of H38 residue in the BaPadR was highlight in yellow.

[b] Green sequence in BaP_{PadC}-RBS represents the engineered RBS sequence (in bold) along with the inserted E.coRI sequence.

[c] The BaPadR binding box1 (BaPadR-1) was highlighted in blue. The BaPadR binding box 2 (BaPadR-2) was highlighted in green.

[d] Deep gray highlight represents shows the entire (original) BaPadR bind box, but when overlapping with the -35 or -10 region, the corresponding sequence was replaced with the -35 or -10 region of the pL_{lacO1} promoter, respectively.

[e] The base replacement on C6 and T8 sites of the BaPadR-1 binding box on the BaP_{padC} promoter and Phy12 promoter were highlighted in red.

[f] The base replacement on T18' site of the BaPadR-1 binding box on the BaP_{padC} promoter and Phy12 promoter were highlighted in violet. The mutations occurred on the complementary chains.

Reference

(1) Atsumi, S.; Cann, A. F.; Connor, M. R.; Shen, C. R.; Smith, K. M.; Brynildsen, M. P.; Chou, K. J. Y.; Hanai, T.; Liao, J. C. Metabolic engineering of Escherichia coli for 1-butanol production. *Metab. Eng.* **2008**, *10* (6), 305-311. DOI: https://doi.org/10.1016/j.ymben.2007.08.003.

(2) Jiang, T.; Li, C.; Zou, Y.; Zhang, J.; Gan, Q.; Yan, Y. Establishing an Autonomous Cascaded Artificial Dynamic (AutoCAD) regulation system for improved pathway performance. *Metab. Eng.* **2022**, *74*, 1-10. DOI: https://doi.org/10.1016/j.ymben.2022.08.009.
